# Cigarette Smoke Extract Impairs Regeneration and Tissue Homeostasis in the Planarian *Dugesia japonica* and May Be Associated with Changes in MAPK-Related Signaling

**DOI:** 10.3390/biology15201821

**Published:** 2026-10-12

**Authors:** Zelong Zhao, Ende Wang, Kexin Yang, Chunmei Zhang, Linxia Song, Zhenbiao Xu

**Affiliations:** 1 Zibo Normal College, Zibo 255100, China; zelong0222@gmail.com; 2 School of Life and Medicine, Shandong University of Technology, Zibo 255049, China; 13280766186@163.com (E.W.); 18632842808@163.com (K.Y.); 19811718781@163.com (C.Z.); slxch@163.com (L.S.)

**Keywords:** cigarette smoke extract, *Dugesia japonica*, oxidative stress, regeneration, MAPK signaling pathway, multi-omics analysis

## Abstract

Cigarette smoke extract (CSE) is used to assess tobacco toxicity, but its effects on freshwater planarian regeneration remain incompletely characterized. We investigated CSE toxicity in *Dugesia japonica* using acute toxicity tests, phenotypic observation, biochemical assays, immunofluorescence, TUNEL staining, and transcriptomic and proteomic analyses. The 96 h median lethal concentration (*LC_50_*) was 8.55% under the study-specific CSE definition. Exposure to 1% and 5% CSE impaired regeneration, with mortality and morphological abnormalities in the 5% group. CSE exposure also altered antioxidant and cholinergic enzyme activities, increased reactive oxygen species (ROS)-related fluorescence and malondialdehyde (MDA) content, and delayed neural remodeling. Fewer H3P-positive mitotic cells and more TUNEL-positive cells were observed, together with changes in proliferation- and apoptosis-related transcripts. Transcriptomic and proteomic analyses identified molecular responses involving stress, metabolism, and cellular homeostasis, although their overall concordance was low. Selected expression changes suggested mitogen-activated protein kinase (MAPK)-related signaling as a candidate response, but pathway activation and causal involvement were not established. These findings characterize regeneration impairment and associated cellular and molecular responses under laboratory CSE exposure conditions. They do not establish ecological risk at environmental exposure levels.

## Simple Summary

Cigarette smoke contains many harmful chemicals, but its effects on tissue regeneration in freshwater animals remain incompletely understood. We exposed planarians, small freshwater flatworms with a strong ability to regrow lost body parts, to a laboratory-prepared cigarette smoke extract. Exposure impaired regeneration and was accompanied by changes in cellular defenses against oxidative damage and in responses related to the nervous system. We also observed fewer dividing cells and more cells with fragmented genetic material. Measurements of gene activity and protein abundance suggested changes in stress responses, energy use, and tissue maintenance. Some findings pointed to a possible role for a cell signaling pathway, but they did not establish that this pathway caused the regeneration defects. Together, these observations connect impaired regeneration with cellular and molecular changes under laboratory exposure conditions. They provide a basis for further investigation of how smoke-derived mixtures affect tissue repair.

## 1. Introduction

Tobacco smoking poses a severe threat to global public health [1]. Cigarette smoke (CS) is a complex mixture containing numerous toxic and carcinogenic compounds [2]. Cigarette smoke extract (CSE) is an experimentally prepared mixture used to study selected biological effects of smoke exposure [3]. Previous studies have demonstrated that CSE induces oxidative stress and apoptosis in mammalian cells [4]. However, its effects on freshwater invertebrates with high regenerative capacity remain incompletely understood.

Planarians (*Dugesia japonica*), which belong to the phylum Platyhelminthes, are ideal model organisms for toxicological and regenerative biology research, owing to their extraordinary regenerative capacity, simple body structure, and high sensitivity to chemical substances [5]. Their regeneration depends on pluripotent adult stem cells termed neoblasts, which account for 20–30% of total somatic cells and can differentiate into all tissue types [6,7]. Planarians have been successfully applied to assess the toxicity of heavy metals, pesticides, microplastics, and pharmaceutical compounds, as their regenerative capacity, oxidative stress response, and gene expression profiles are highly sensitive to environmental pollutants [8,9,10]. Notably, stem cells from aquatic invertebrates have emerged as advanced tools for ecotoxicological impact assessment, rendering planarians particularly valuable for investigating the toxic effects of CSE on stem cell function and tissue regeneration [11].

Although CSE toxicity has been investigated in several biological models, few studies have evaluated its effects on planarians at the whole-organism, biochemical, cellular, and molecular levels. Previous studies have reported impaired tissue regeneration in zebrafish and oxidative stress in mammalian cells after cigarette-smoke-related exposure [4,12]. The effects of CSE on planarian regeneration and associated molecular responses remain incompletely understood. Transcriptomic and proteomic analyses can identify changes in genes and proteins and help generate hypotheses about the pathways involved.

In this study, we aimed to investigate the effects of CSE on *D. japonica* and characterize associated molecular responses. We first determined the 96 h median lethal concentration (*LC_50_*) of CSE. We then evaluated regeneration, oxidative stress-related indicators, cholinergic enzyme activity, neural remodeling, cell proliferation-related indicators, and apoptosis-related indicators. Transcriptomic and proteomic analyses were used to explore molecular responses, followed by targeted analysis of selected stress-response genes and proteins. MAPK-related signaling was considered a candidate response rather than an established mechanism. The study was designed to characterize toxicity under controlled laboratory exposure conditions.

## 2. Materials and Methods

### 2.1. Materials

Hongqiqu brand cigarettes (10 mg tar, 0.9 mg nicotine, 11 mg CO per cigarette) were purchased from Henan Tobacco Industrial Co., Ltd., Zhengzhou, China, and used for CSE preparation without filter tip removal unless otherwise specified. Key reagents included TRIzol reagent (Invitrogen, Carlsbad, CA, USA); Reverse Transcription Kit (Takara Bio Inc., Otsu, Japan); TB Green Premix Ex Taq II (Takara Bio Inc., Otsu, Japan); BCA Protein Assay Kit (A045-4-2), SOD (A001-3-2), CAT (A007-1-2), ChAT (A079-1-1), and AChE (A024-1-1) activity assay kits and MDA assay kit (A003-1-2; Nanjing Jiancheng Bioengineering Institute, Nanjing, China); ROS detection kit (Wuhan Servicebio Technology Co., Ltd., Wuhan, China); One Step TUNEL Apoptosis Assay Kit (Green Fluorescence; C1088; Beyotime Biotechnology, Shanghai, China); anti-phospho-histone H3 (Ser10; H3P) mouse monoclonal antibody (ab14955; Abcam, Cambridge, UK); mouse monoclonal anti-synapsin antibody (clone 3C11, anti-SYNORF1; RRID: AB_528479; Developmental Studies Hybridoma Bank, University of Iowa, Iowa City, IA, USA); and primary antibodies against pan-RAS (81615-1-PBS), HSP70 (66183-1-Ig), and IL-8/CXCL8 (31533-1-AP) and cleaved caspase-3 (82707-13-RR; Proteintech/Wuhan Sanying Biotechnology Co., Ltd., Wuhan, China). Other reagents included FITC-conjugated goat anti-mouse IgG (A0568), Cy3-conjugated goat anti-mouse IgG (A0521), SDS-PAGE Sample Loading Buffer (5X; P0015), and BeyoECL Star (P0018AS; Beyotime Biotechnology, Shanghai, China); and polymer HRP-conjugated goat anti-rabbit IgG (H+L) (RGAR011) and goat anti-rabbit/mouse IgG (H+L) (RGAU011; Proteintech/Wuhan Sanying Biotechnology Co., Ltd., Wuhan, China). Major instruments included a gas chromatography–mass spectrometry (GC-MS) system (Agilent 7000D, Agilent Technologies Inc., Santa Clara, CA, USA), a real-time fluorescence quantitative PCR system (LightCycler 480 II, Roche Diagnostics, Basel, Switzerland), a fluorescence imaging system equipped with a DS-Fi3 camera (Nikon, Tokyo, Japan), a Western blot imaging system (SH-Advance 523, Hangzhou Shenhua Technology Co., Ltd., Hangzhou, China), a high-throughput sequencing platform (Illumina NovaSeq 6000, Illumina Inc., San Diego, CA, USA), and an Orbitrap Astral high-resolution mass spectrometer (Thermo Fisher Scientific, Waltham, MA, USA).

### 2.2. Experimental Animals

An asexual strain of *Dugesia japonica* (ZB-1) was maintained in our laboratory. Planarians were cultured in Montjuïc water (1.6 mmol L^−1^ NaCl, 1.0 mmol L^−1^ CaCl_2_, 1.0 mmol L^−1^ MgSO_4_, 0.1 mmol L^−1^ MgCl_2_, 0.1 mmol L^−1^ KCl, and 1.2 mmol L^−1^ NaHCO_3_) at 20 ± 1 °C in a biochemical incubator (SPX-2508SH, Shanghai CIMO Medical Instrument Manufacturing Co., Ltd., Shanghai, China) [13]. They were fed nematode homogenate once a week and starved for 7 days before experiments to minimize the influence of recent feeding. Planarians of a uniform size (approximately 5 mm in length) and with intact morphology were selected for all experiments. A total of 1290 individual animals were used in the formal experiments, and more than 1650 were used when preliminary concentration screening and methodological trials were included. Each regeneration fragment was counted by its donor animal. Repeated observations and RNA–protein aliquots from the same pool were not counted as additional animals. Transcriptomic and proteomic analyses shared the same animal pools; the other experimental assays used separate batches.

### 2.3. Preparation of Cigarette Smoke Extract (CSE)

**Figure A1:**
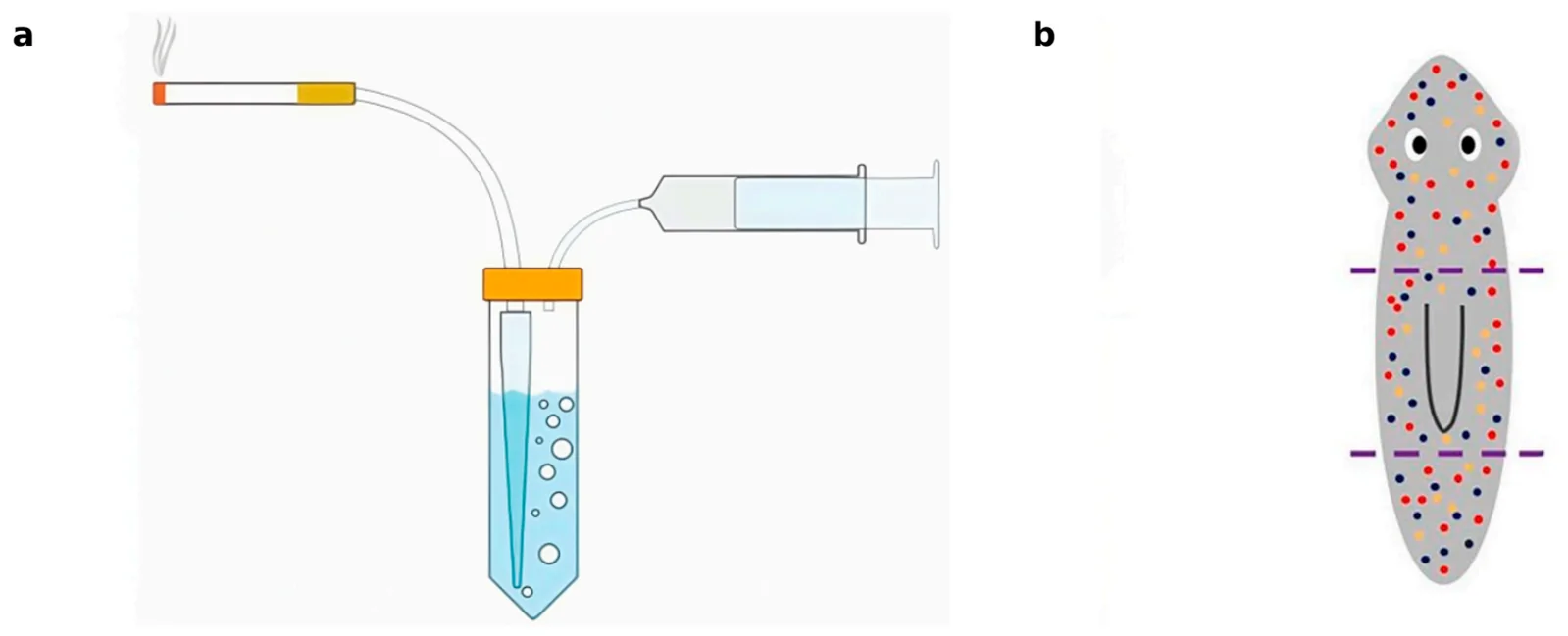
Schematic diagram of CSE preparation and planarian amputation for regeneration assay. Note: (**a**) Preparation procedure of cigarette smoke extract; (**b**) amputation sites for planarian regeneration experiment.

CSE was prepared using an aqueous smoke-extraction approach [3], with study-specific conditions described below. Absorbance-based normalization of CSE preparations has also been used in previous studies [14,15]. Briefly, a single Hongqiqu cigarette was burned, and the smoke was introduced into 10 mL of Montjuïc water using a modified syringe-driven device over 5 min (Figure A1a). The absorbance of the resulting solution was measured at 320 nm using a microplate reader (Synergy HTX, BioTek Instruments, Winooski, VT, USA). The solution was adjusted with Montjuïc water to an empirically selected, study-specific absorbance target of 0.50, with a predefined allowable range of 0.48–0.52, and defined operationally as 100% CSE. Montjuïc water was the extraction and dilution medium and the animal-exposure control; no organic solvent was added to the exposure preparations. Working preparations (1%, 2%, and 5%) were obtained by dilution of this stock, freshly prepared before use, and used within 30 min. For exposures lasting up to 7 days, the working solution was completely renewed every 24 h using working solutions diluted from a new CSE stock prepared that day and meeting the absorbance criterion. The allowable range is a predefined preparation criterion, not a measured standard deviation or estimate of between-batch variation. These percentages describe dilution relative to the operationally defined stock; they do not represent measured concentrations of individual smoke constituents. 

### 2.4. GC-MS Analysis of CS and CSE

**Figure 1:**
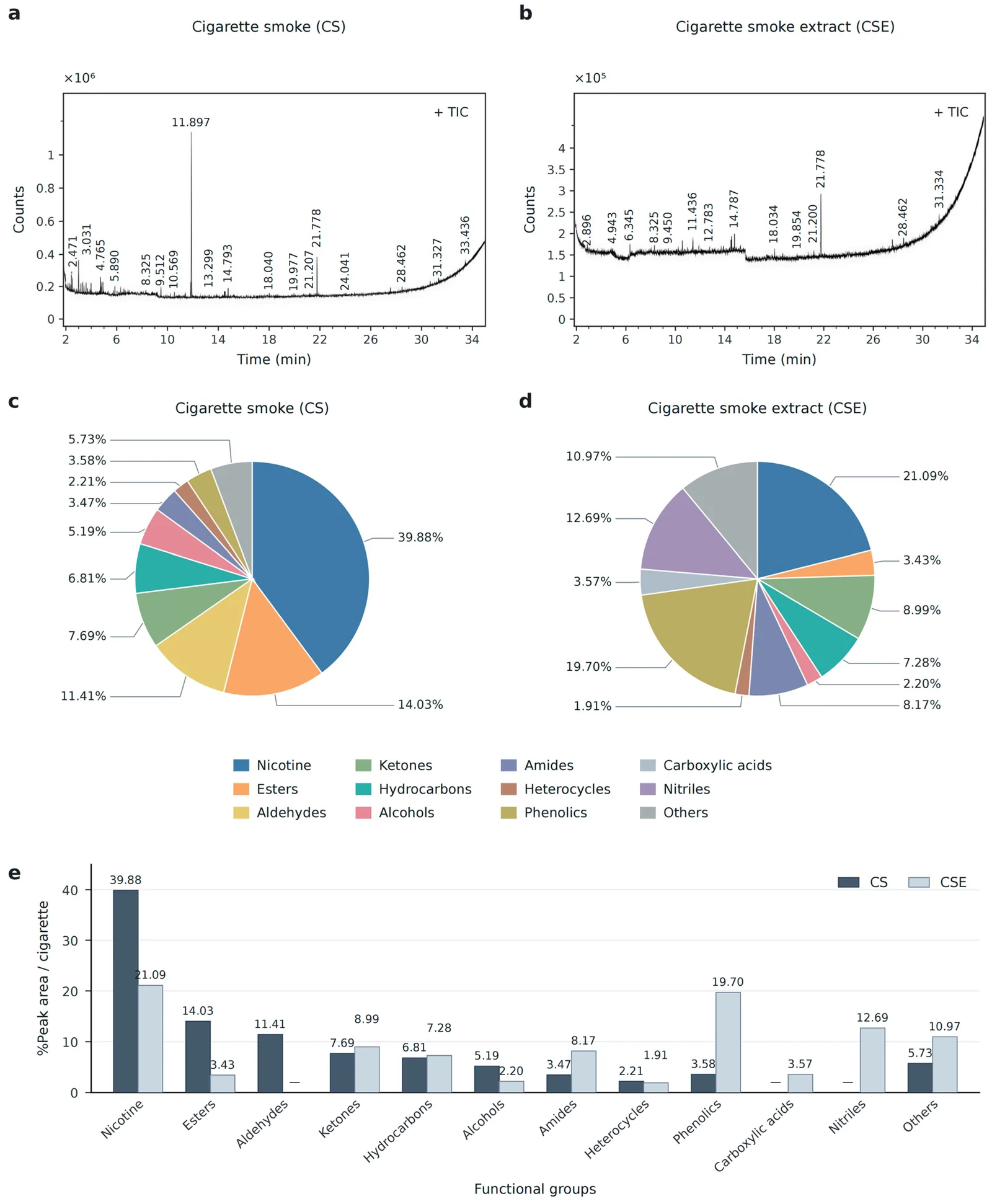
Component analysis of cigarette smoke (CS) and cigarette smoke extract (CSE) by GC-MS. Note: (**a**) Total ion chromatogram of cigarette smoke; (**b**) total ion chromatogram of cigarette smoke extract; (**c**) relative peak-area profile of detected chemical components in cigarette smoke; (**d**) relative peak-area profile of detected chemical components in cigarette smoke extract; (**e**) descriptive summary of relative peak areas by functional group for each preparation. CS and CSE were analyzed using different preparation and injection methods; relative peak areas are not compound concentrations and should not be compared quantitatively between preparations.

The components of CS and CSE were analyzed by GC-MS following the method of Kim et al. [16] with modifications. For CSE analysis, a separate 10 mL aliquot was extracted with 2 mL of dichloromethane, vortexed thoroughly, and separated into organic and aqueous phases. The organic phase was filtered through a 0.22 μm membrane and injected into the GC-MS system. This extraction was performed for chemical analysis only; the dichloromethane extract was not used for animal exposure. For CS analysis, smoke from a burning cigarette was collected in a gas collection bottle filled with helium and analyzed by headspace injection (heating at 85 °C for 15 min). The GC column was a DB-Wax capillary column (30 m × 0.25 mm × 0.25 μm), with helium as the carrier gas (flow rate: 1 mL min^−1^). The injection temperature was 250 °C, and the oven temperature program was as follows: initial temperature 40 °C (held for 3 min), increased to 220 °C at 5 °C min^−1^ (held for 10 min). The MS detector was operated in electron ionization mode (70 eV), and the mass scan range was m/z 35–500. Data were processed using Thermo Xcalibur v4.2 software, and compounds were identified by matching with the NIST library. Relative peak-area proportions were calculated by peak-area normalization within each preparation (Figure 1). These proportions describe the detected signal profile and are not mass fractions or constituent concentrations. Because CS and CSE underwent different preparation and injection procedures, their relative peak areas were not used to infer quantitative enrichment or depletion between preparations.

### 2.5. Acute Toxicity Assay

The 96 h median lethal concentration (*LC_50_*) of CSE to *D. japonica* was determined using an acute toxicity test. Based on preliminary experiments, six CSE concentrations (6.0%, 7.0%, 8.0%, 9.0%, 10.0%, and 11.0%) were tested. Planarians (10 animals per group in each of three independent exposure batches) were randomly assigned to each concentration group, with Montjuïc water as the control. The culture medium was renewed every 24 h, and the number of dead planarians (judged by body disintegration) was recorded at 24, 48, 72, and 96 h. The *LC_50_* was estimated using the probit link in SPSS 26.0, with deaths and the number exposed at each concentration as the response inputs [17]. The probit link uses the inverse standard normal cumulative distribution function; the *LC_50_* corresponds to a fitted death probability of 0.5. The 95% limits were taken from the SPSS PROBIT confidence-interval output, termed fiducial confidence limits in the software documentation [18]. Each batch used independently prepared exposure solutions; batch-level outcomes were used for statistical summaries.

### 2.6. Regeneration Assay

**Figure 2:**
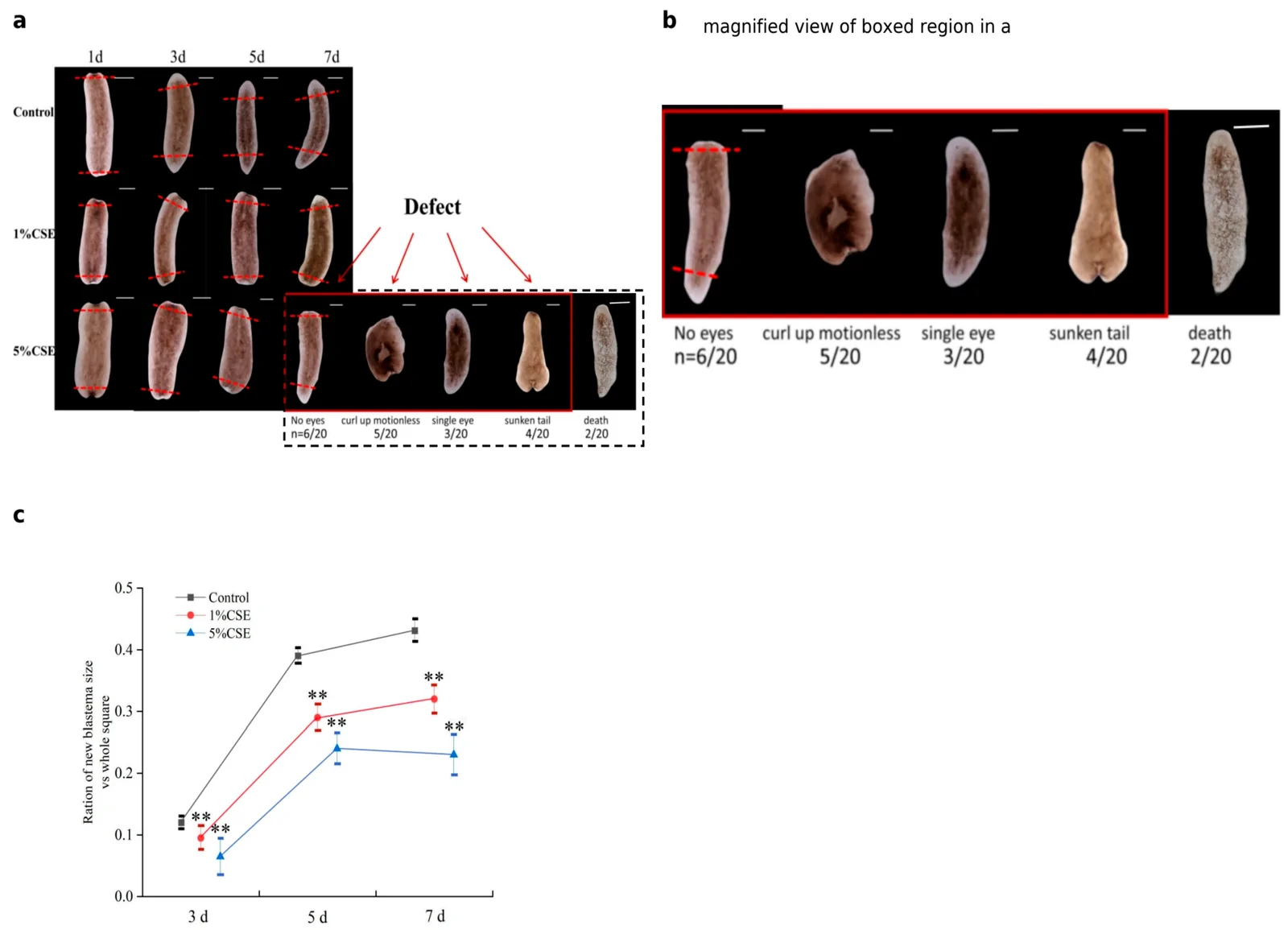
Effects of cigarette smoke extract on planarian regeneration. Note: (**a**) Regeneration phenotypes of planarians at 1, 3, 5, and 7 days post-amputation under different concentrations of CSE. (**b**) Examples of phenotypic defects and mortality in the 5% CSE group, with the displayed category frequencies reported descriptively. (**c**) Quantification of the blastema-to-total-body-area ratio (s/S) at 3, 5, and 7 days post-amputation, where s is projected blastema area, and S is the total projected body area of the same specimen at the same time. The ratio is a relative-area index, not an absolute area measurement. In panel (**c**), symbols indicate statistically significant differences at *p* < 0.01 (**). Each condition included 20 fragments per batch, with three independent exposure batches. Scale bar = 500 µm. The area-ratio comparisons in panel (**c**) used one-way ANOVA followed by Tukey’s HSD. Concentration groups were compared separately at each sampling time, using the independent batch summaries (*n* = 3).

Planarians were transversely cut at sites anterior to the auricles and posterior to the pharynx to obtain anterior, middle, and posterior fragments. The middle fragments (20 fragments per group in each of three independent exposure batches) were exposed to 1% and 5% CSE, with Montjuïc water as the control. Fragments were randomly assigned to the exposure groups, with each group housed together in one well of a six-well plate in each batch. The same groups of fragments were followed over time, and batch-level summaries were used for statistical analysis. Regeneration phenotypes were observed and photographed under a stereomicroscope (SZ650, Chongqing Aotai Optical Instrument Co., Ltd., Zhengzhou, China) at 1, 3, 5, and 7 days post-amputation (Figure A1b). The area of the regenerative blastema (s) and total body area (S) were measured using ImageJ v1.53t [19], and the regeneration index was calculated from the ratio s/S. Both areas refer to the same specimen at the same observation time. Figure 2c displays this dimensionless ratio; multiplying it by 100 gives the corresponding percentage. This relative two-dimensional area index has also been used in planarian regeneration research [20]; it describes the proportion of body area occupied by the blastema, rather than absolute new-tissue growth. Exploratory preliminary screening at 0.5%, 1%, 2%, 5%, and 7% CSE considered regeneration, H3P labeling, TUNEL labeling, and survival. These qualitative observations informed concentration selection and were not a complete, replicated dose–response dataset. The 1% and 5% preparations were selected as lower and higher exposure conditions with observable responses. Although both were below the 96 h *LC_50_*, the 5% condition was not nonlethal.

### 2.7. Biochemical Assays

Planarians (10 animals pooled per biological sample; three independent exposure batches per condition) were exposed to 1%, 2%, and 5% CSE for 1, 3, and 5 days. Each group was exposed together in a well of a six-well plate, with 10 animals per group per batch; these animals were pooled for one biochemical sample. Total proteins were extracted using RIPA lysis buffer containing protease inhibitors, and the protein concentration was determined by the BCA method using kit A045-4-2 (Nanjing Jiancheng Bioengineering Institute, Nanjing, China) [21,22]. Each biological replicate underwent independent exposure, protein extraction, and measurement. Each pooled sample was assayed once without additional technical replicates.

The activities of SOD, CAT, ChAT, and AChE, and MDA content were measured with the corresponding Nanjing Jiancheng kits according to the manufacturer’s protocols. BCA protein measurement (A045-4-2) used the protein-dependent formation of a copper–BCA complex, read at 562 nm [22]. Protein concentration was calculated as Cprotein = [(Asample − Ablank)/(Astandard − Ablank)] × Cstandard × NBCA, where Cstandard is the supplied protein-standard concentration, and NBCA is the dilution factor for protein measurement. Protein concentrations were expressed in mg/mL for normalization. In the activity calculations below, A denotes absorbance, Cpr is the protein concentration (mg/mL) of the normalization sample, and N is any additional assay dilution relative to that sample; N = 1 when no additional dilution is used.

**Figure 3:**
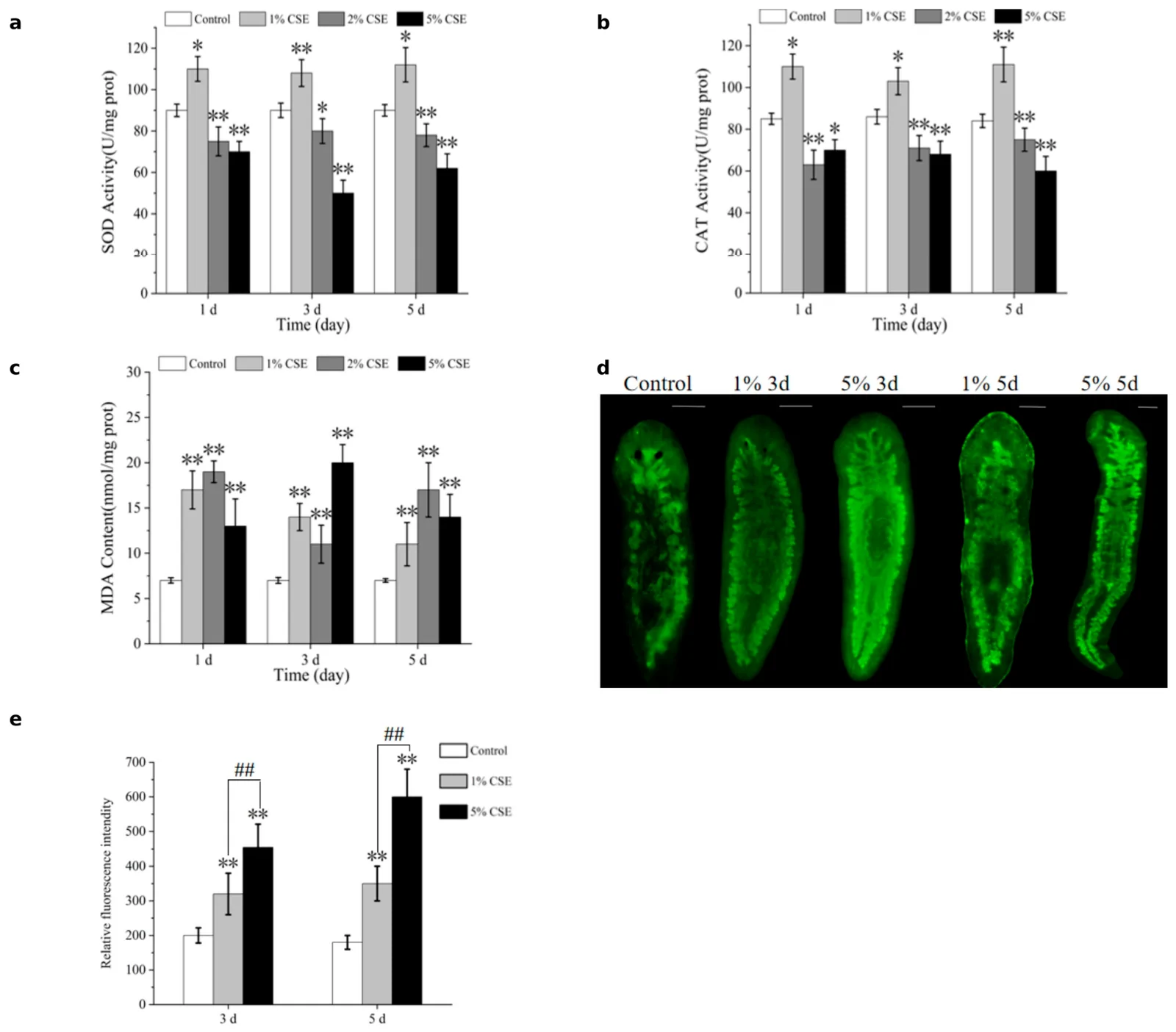
Effects of cigarette smoke extract on antioxidant enzyme activities and oxidative stress levels in planarians. Note: (**a**) Superoxide dismutase (SOD) activity. (**b**) Catalase (CAT) activity. (**c**) Relative malondialdehyde (MDA) content. (**d**) DCFH-DA oxidation-related fluorescence after 3 and 5 days of exposure to 1% and 5% CSE. (**e**) Whole-animal mean fluorescence intensity in arbitrary units, summarized by independent exposure batch. The water-exposed group at the same time and in the same batch was the comparison reference. The signal does not identify a specific reactive oxygen species or its concentration. Symbols indicate statistically significant differences at *p* < 0.05 (*) or *p* < 0.01 (** or ##). Scale bar = 500 µm. Multiple-group comparisons used one-way ANOVA followed by Tukey’s HSD; comparisons involving two independent groups used independent-samples *t*-tests. Concentration groups were compared separately at each sampling time, using the independent batch summaries (*n* = 3).

SOD activity was measured by inhibition of superoxide-dependent WST-1 reduction using kit A001-3-2 [23,24]. The reaction contained 20 μL of sample in a total volume of 240 μL, was incubated at 37 °C for 20 min, and was read at 450 nm. The fractional inhibition was I = 1 − (Atest − Atest blank)/(Acontrol − Acontrol blank). The control contained the superoxide-generating reaction without sample, while the corresponding blanks omitted the enzyme working solution. Activity was calculated as SOD (U/mg protein) = (I/0.50) × (240/20) × N/Cpr. One kit unit corresponds to the amount of enzyme producing 50% inhibition under the assay conditions (Figure 3a).

CAT activity was measured using the visible-light microplate kit A007-1-2, based on hydrogen peroxide decomposition [25,26]. Activity was calculated using the kit’s activity conversion and normalized as CAT (U/mg protein) = CCAT × N/Cpr, where CCAT is the activity concentration (U/mL) obtained for the assayed sample using the kit calculation. The kit-specific reaction conditions and unit conversion were used for this colorimetric endpoint (Figure 3b).

**Figure 4:**
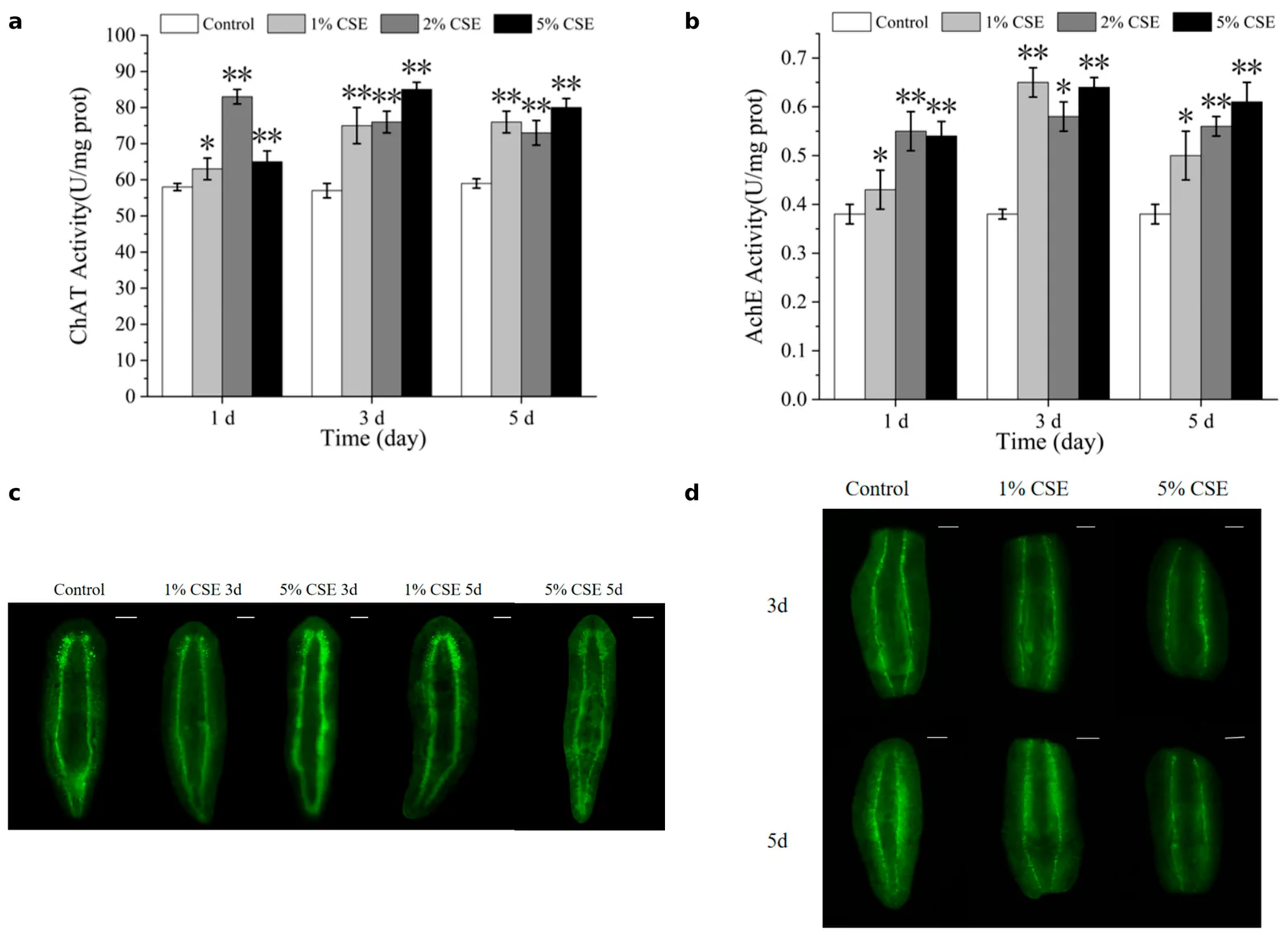
Effects of cigarette smoke extract on cholinergic enzyme activities and neural structure in planarians. Note: (**a**) Choline acetyltransferase (ChAT) activity; (**b**) acetylcholinesterase (AChE) activity; (**c**) nervous system structure of intact planarians detected by synapsin immunofluorescence; (**d**) nervous system structure of regenerating planarians detected by synapsin immunofluorescence. The primary antibody in panels (**c**,**d**) was mouse monoclonal anti-synapsin (clone 3C11, anti-SYNORF1; DSHB; RRID: AB_528479; 1:200). Symbols indicate statistically significant differences at *p* < 0.05 (*) or *p* < 0.01 (**). Scale bar = 500 µm. Multiple-group comparisons used one-way ANOVA followed by Tukey’s HSD; comparisons involving two independent groups used independent-samples *t*-tests. Concentration groups were compared separately at each sampling time, using the independent batch summaries (*n* = 3).

ChAT activity was determined using kit A079-1-1. ChAT transfers the acetyl group from acetyl-CoA to choline, producing acetylcholine and coenzyme A [27]. The kit detects the associated chromogenic reaction at 324 nm [28]. A boiled sample preparation served as the matched reaction control. After the 37 °C, 20 min enzymatic reaction and the kit’s stopping and color-development steps, activity was calculated as ChAT (U/mg protein) = (Atest − Acontrol) × Vr × N/(t × k × l × Vs × Cpr). Here, t = 20 min, Vr = 600 μL, Vs = 25 μL, l = 1 cm, and k = 1.98 × 10^−2^ is the conversion coefficient specified in the kit instructions. The result follows the kit’s protein-normalized unit convention (Figure 4a).

AChE activity was measured with kit A024-1-1 by colorimetric detection of enzyme-dependent substrate hydrolysis at 412 nm [29,30]. A matched sample control received the sample after addition of the inhibitor. The reaction was conducted at 37 °C for 6 min. Activity was calculated as AChE (U/mg protein) = [(Atest − Acontrol)/(Astandard − Ablank)] × Cstandard × N × (6/t)/Cpr, where Cstandard = 1 μmol/mL, and t is the reaction time in minutes. The kit defines one unit by hydrolysis of 1 μmol of substrate during 6 min under its specified conditions; this six-minute convention was retained (Figure 4b).

MDA was measured with the TBA kit A003-1-2. The reaction forms a colored MDA–TBA product after heating at 95 °C for 40 min; the clarified reaction mixture was read at 532 nm [31]. MDA content (nmol/mg protein) = [(Atest − Acontrol)/(Astandard − Ablank)] × Cstandard × N/Cpr, where Cstandard = 10 nmol/mL. The sample control omits the TBA-containing reagent; the kit permits the reagent blank to substitute for this control when sample color and turbidity do not contribute appreciable background (Figure 3c).

For ROS detection, planarians (10 animals per group in each of three independent exposure batches) were exposed to 1% and 5% CSE for 3 and 5 days, stained with DCFH-DA probe (10 μmol L^−1^) at room temperature for 2 h in the dark, rinsed three times with culture buffer, and imaged immediately under a fluorescence microscope. Excitation/emission settings, exposure time, and instrument gain were kept the same across groups. Whole-animal regions of interest were outlined in ImageJ, and mean fluorescence intensity within each region was measured [19,32]. The mean intensity is the summed pixel intensity divided by the number of pixels in the region. Measurements were summarized within each independent exposure batch. The Montjuïc-water group at the same sampling time and in the same batch served as the comparison reference (Figure 3d).

### 2.8. Gene Expression Analysis

**Figure 5:**
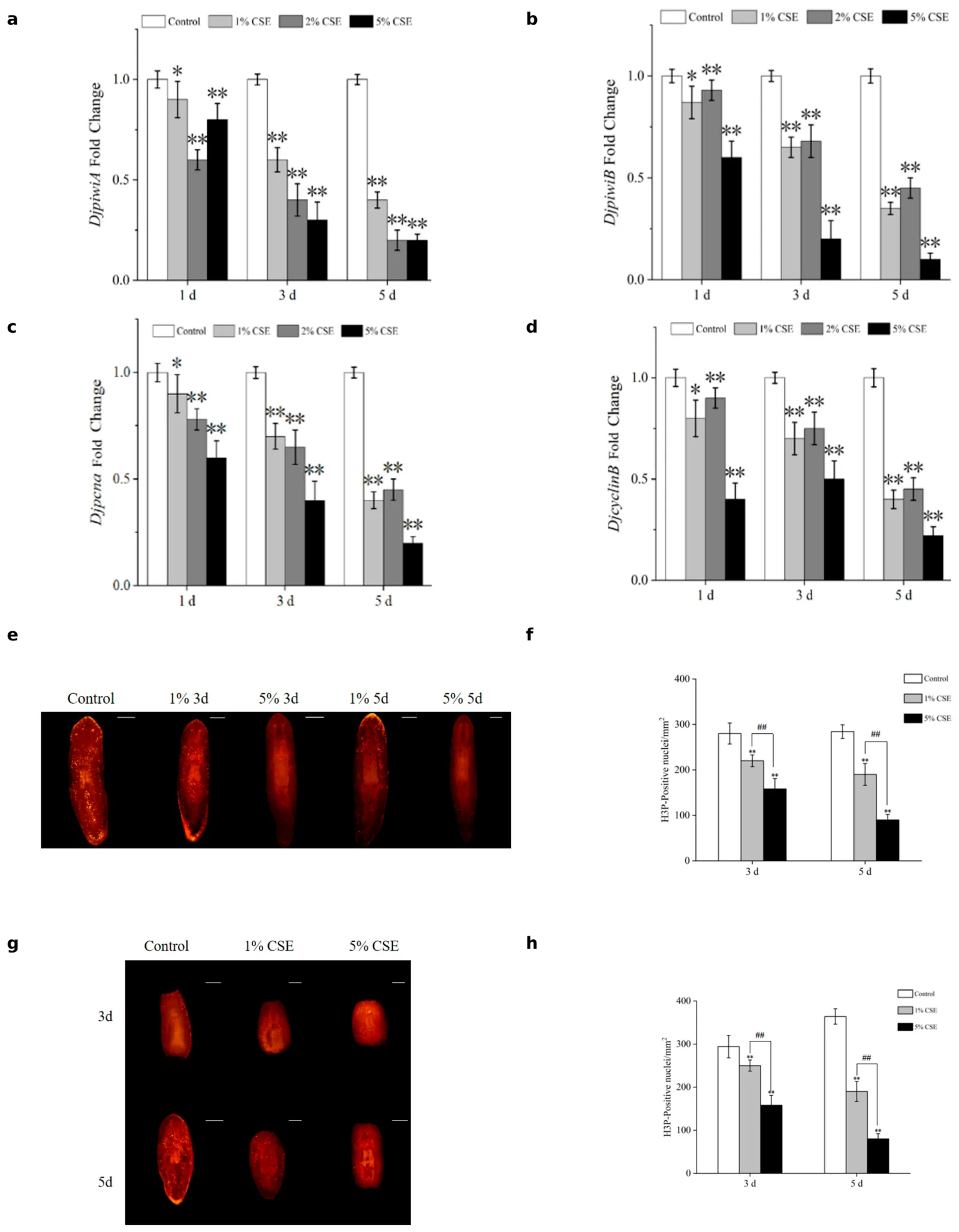
Effects of cigarette smoke extract on proliferation-related gene expression and mitotic cell abundance in planarians. Note: (**a**) Relative expression of *DjpiwiA*; (**b**) relative expression of *DjpiwiB*; (**c**) relative expression of *Djpcna*; (**d**) relative expression of *DjCyclin B*; (**e**) fluorescence detection of H3P^+^ mitotic cells in intact planarians; (**f**) quantification of H3P^+^ cells in intact planarians; (**g**) fluorescence detection of H3P^+^ mitotic cells in regenerating planarians; (**h**) quantification of H3P^+^ cells in regenerating planarians. Panels (**a**–**d**) show qPCR after 1, 3, and 5 days of exposure to 1%, 2%, and 5% CSE. Panels (**e**–**h**) show H3P staining after 3 and 5 days of exposure to 1% and 5% CSE, using anti-phospho-histone H3 (Ser10; Abcam, ab14955; 1:200). Values in panels (**f**,**h**) are positive nuclei per mm^2^. Symbols indicate statistically significant differences at *p* < 0.05 (*) or *p* < 0.01 (** or ##). Scale bar = 500 μm. Multiple-group comparisons used one-way ANOVA followed by Tukey’s HSD; comparisons involving two independent groups used independent-samples *t*-tests. Concentration groups were compared separately at each sampling time, using the independent batch summaries (*n* = 3).

**Figure 6:**
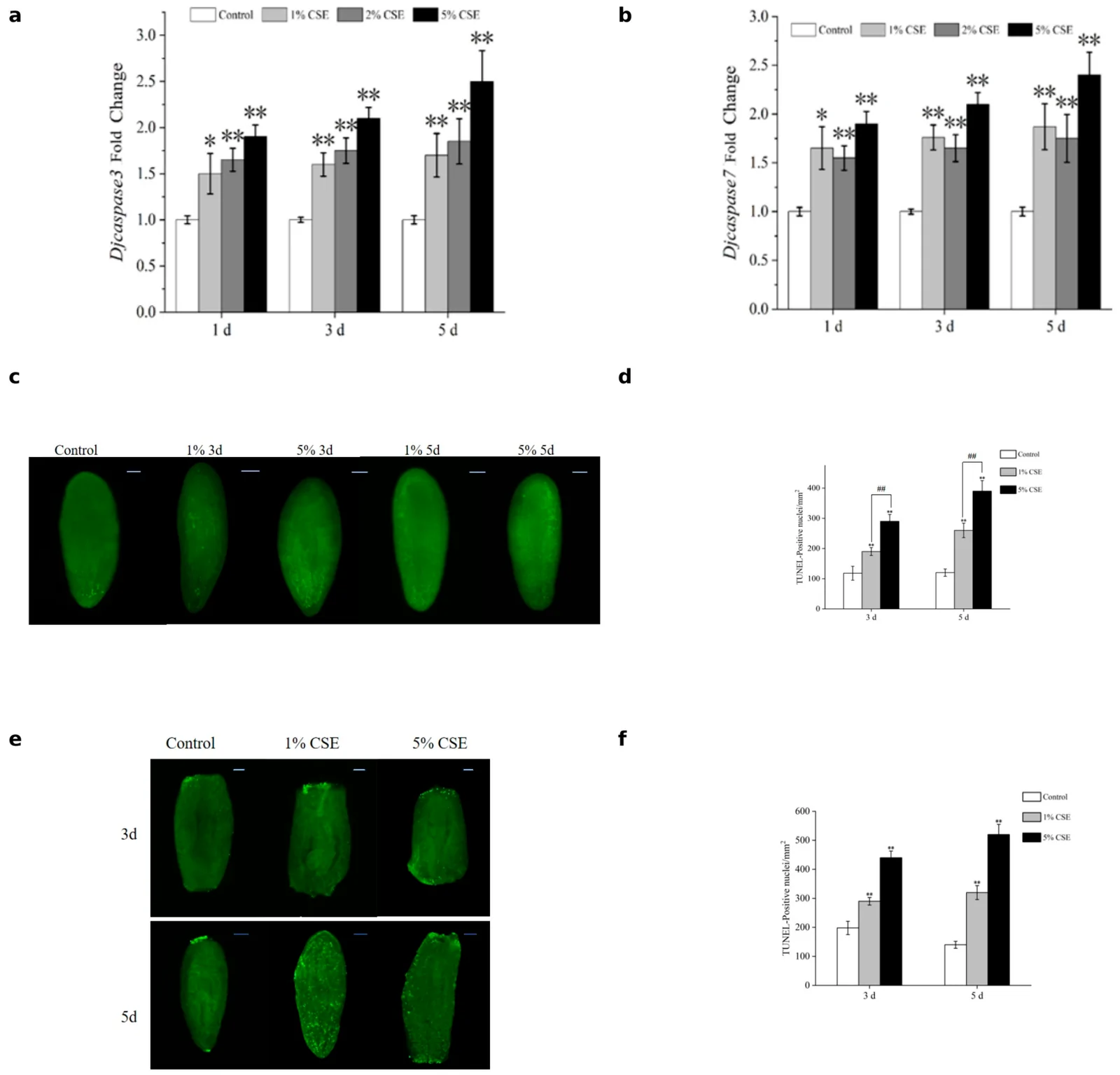
Effects of cigarette smoke extract on apoptosis-related gene expression and TUNEL labeling in planarians. Note: (**a**) Relative expression of *DjCaspase3*; (**b**) relative expression of *DjCaspase7*; (**c**) fluorescence detection of TUNEL^+^ cells in intact planarians; (**d**) quantification of TUNEL^+^ cells in intact planarians; (**e**) fluorescence detection of TUNEL^+^ cells in regenerating planarians; (**f**) quantification of TUNEL^+^ cells in regenerating planarians. Panels (**a**,**b**) show qPCR after 1, 3, and 5 days of exposure to 1%, 2%, and 5% CSE. Panels (**c**–**f**) show TUNEL labeling after 3 and 5 days of exposure to 1% and 5% CSE; values in panels (**d**,**f**) are positive nuclei per mm^2^. Symbols indicate statistically significant differences at *p* < 0.05 (*) or *p* < 0.01 (** or ##). Scale bar = 500 μm. Multiple-group comparisons used one-way ANOVA followed by Tukey’s HSD; comparisons involving two independent groups used independent-samples *t*-tests. Concentration groups were compared separately at each sampling time, using the independent batch summaries (*n* = 3).

For proliferation- and apoptosis-related qPCR (Figure 5a–d and Figure 6a,b), samples were collected after 1, 3, and 5 days of exposure to 1%, 2%, and 5% CSE, with Montjuïc water as the control. Sampling for selected stress-response transcripts is specified in Section 2.11. Total RNA was extracted from planarians (10 animals pooled per biological sample; three independent biological replicates per condition) using TRIzol reagent (Invitrogen, Carlsbad, CA, USA) following the manufacturer’s instructions. RNA quality assessment used a NanoPhotometer NP80 (Implen GmbH, Munich, Germany) and an Agilent 2100 Bioanalyzer (Agilent Technologies, Santa Clara, CA, USA), with stated acceptance criteria of OD_260_/OD_280_ = 1.8–2.0 and RIN > 7.0. cDNA was synthesized from 2 μg of total RNA using a Reverse Transcription Kit (Takara Bio Inc., Otsu, Japan) with oligo(dT) primers. Exposure, RNA extraction, and cDNA preparation were performed independently for each biological replicate. For these pooled qPCR samples, each exposure group contained 10 animals housed together in a well of a six-well plate per batch; the 10 animals were pooled for each RNA sample.

Primers for qPCR were designed using Primer 5.0 software (Premier Biosoft International, Palo Alto, CA, USA) based on the transcriptome sequencing data, with *β-DjActin* as the internal reference gene. The primer sequences are listed in Table A1, and all primers were synthesized by Beijing Huada Gene Technology Co., Ltd., Beijing, China. qPCR was performed on a LightCycler 480 II instrument (Roche, Basel, Switzerland) using TB Green Premix Ex Taq II (Takara Bio Inc., Otsu, Japan). The reaction system (10 μL) contained 1 μL cDNA (10-fold diluted), 5 μL SYBR Green mix, 0.4 μL forward primer (10 μmol L^−1^), 0.4 μL reverse primer (10 μmol L^−1^), and 3.2 μL ddH_2_O. The reaction program was: 95 °C for 30 s; 40 cycles of 95 °C for 5 s and 60 °C for 30 s. The relative expression levels of target genes (*DjpiwiA*, *DjpiwiB*, *Djpcna*, *DjCyclin B*, *DjCaspase3*, *DjCaspase7*, *DjMAP3K*, *DjRAS*, *DjHSP70*, *DjIL-8-like*) were calculated using the 2^−ΔΔCt^ method [33], with *β-DjActin* as the internal reference. Each cDNA sample was amplified in technical triplicate. Technical reactions were summarized within each biological sample, and statistical inference used the three independent biological replicates. 

### 2.9. Immunofluorescence and TUNEL Assays

Planarians (10 animals per group in each of three independent exposure batches) were exposed together by group in wells of six-well plates to 1% and 5% CSE for 3 and 5 days. Samples were fixed with 4% paraformaldehyde at 4 °C for 12 h, dehydrated through a methanol gradient, and bleached with 6% H_2_O_2_ for 16 h. After rehydration, samples were blocked with 5% BSA for 4 h, incubated with primary antibodies (anti-H3P, ab14955, 1:200; anti-synapsin, clone 3C11/anti-SYNORF1, 1:200) at 4 °C overnight, and then incubated with fluorescent goat anti-mouse IgG secondary antibodies (Cy3, A0521; FITC, A0568; 1:500) in the dark for 12 h. Samples were washed with PBST (PBS containing 0.1% Tween-20) three times (15 min each) between steps, mounted in 80% glycerol, and observed under a fluorescence microscope. H3P staining was used to identify mitotic cells [34]. The quantitative endpoint in Figure 5f,h is the density of H3P-positive nuclei, expressed as nuclei per mm^2^. Exposure, fixation, staining, imaging, and counting were performed independently for each batch; within-batch measurements were summarized before statistical analysis. Preliminary H3P staining included a primary-antibody-omission control, with the subsequent secondary-antibody staining retained; no obvious specific fluorescence was observed. This assessed staining background rather than independently establishing antigen specificity.

DNA fragmentation associated with apoptosis was assessed using a One Step TUNEL Apoptosis Assay Kit (Green Fluorescence; C1088; Beyotime Biotechnology, China) [35,36]. In this assay, terminal deoxynucleotidyl transferase (TdT) incorporates fluorescein-labeled dUTP at accessible 3′-OH DNA ends [36,37,38]; the reaction is distinct from antibody-based immunofluorescence. Fixed planarians were permeabilized with 1% SDS for 20 min, washed with PBST, and incubated with freshly prepared TUNEL reaction mixture (TdT enzyme solution:labeling solution, 1:9 by volume) at 37 °C for 3 h in the dark. This incubation was the whole-mount condition used in this study. After washing, samples were mounted and observed under a fluorescence microscope. A negative control was included during preliminary TUNEL staining with this kit. TUNEL-positive nuclei were counted using ImageJ software; the quantitative endpoint in Figure 6d,f is positive nuclear density, expressed as nuclei per mm^2^. TUNEL staining was interpreted together with the other apoptosis-related measurements. Three independent exposure and staining batches were used per condition, with 10 animals per group housed together in a well of a six-well plate in each batch. Within-batch counts were summarized, with the independent batch as the unit of statistical inference.

### 2.10. Transcriptome and Proteome Sequencing

**Figure 7:**
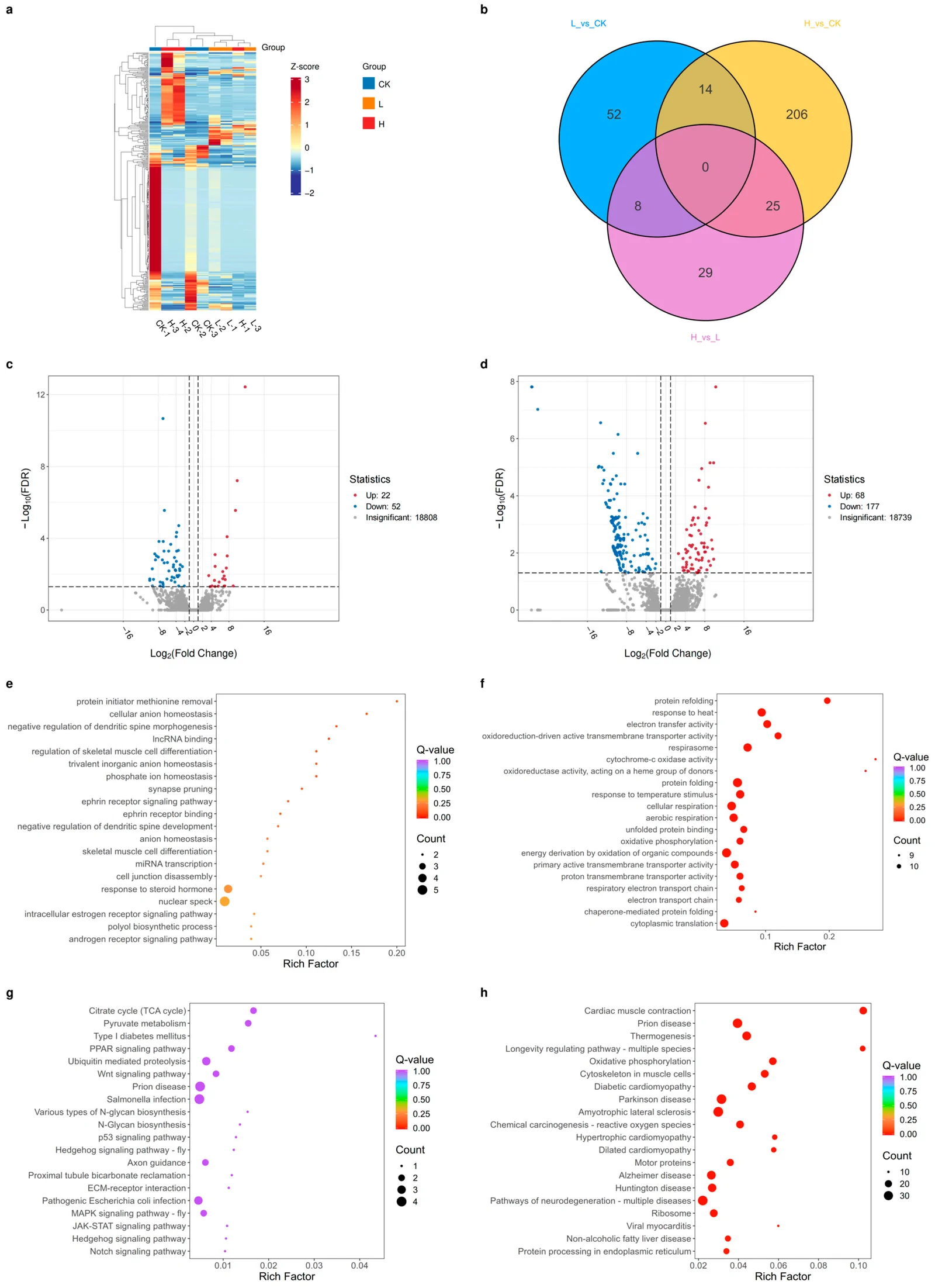
Transcriptomic profiling of planarians exposed to cigarette smoke extract. Note: (**a**) Hierarchical clustering heatmap of differentially expressed genes (DEGs) across samples. (**b**) Venn diagram of DEGs in L vs. CK, H vs. CK, and H vs. L. Each total includes every region within its circle: L vs. CK, 52 + 14 + 8 + 0 = 74; H vs. CK, 206 + 14 + 25 + 0 = 245; H vs. L, 29 + 8 + 25 + 0 = 62. The regions labeled 14, 8, and 25 are pairwise-only intersections; the central three-way intersection contains 0 genes. (**c**) Volcano plot of DEGs between the control (CK) group and the 1% CSE (L) group. (**d**) Volcano plot of DEGs between the CK group and the 5% CSE (H) group. (**e**) GO enrichment analysis of DEGs (Control vs. 1% CSE). (**f**) GO enrichment analysis of DEGs (Control vs. 5% CSE). (**g**) KEGG pathway enrichment analysis of DEGs (Control vs. 1% CSE). (**h**) KEGG pathway enrichment analysis of DEGs (Control vs. 5% CSE). The volcano plots display −log_10_(FDR), and the enrichment panels display Q values; inclusion of a term in a plot does not by itself establish significant enrichment.

Planarians (10 animals pooled per biological sample) were exposed to 1% and 5% CSE for 72 h, with Montjuïc water as the control (3 biological replicates per group). Each biological replicate originated from a separately prepared and independently exposed batch. Within each batch, the 10 animals per condition were pooled and homogenized; the homogenate was divided into two equal aliquots for RNA and protein extraction, respectively. Thus, the transcriptomic and proteomic datasets were paired at the pooled-sample level and shared the same three biological replicates per condition. Aliquots did not constitute additional biological replicates. Total RNA was extracted. RIN values for the nine transcriptomic RNA samples ranged from 7.2 to 8.3 and were retrieved from laboratory notebook records. cDNA libraries were constructed using the NEBNext^®^ Ultra™ RNA Library Prep Kit for Illumina^®^ (New England Biolabs, Ipswich, MA, USA). Libraries were sequenced on an Illumina NovaSeq 6000 platform, generating 150 bp paired-end reads. Raw reads were filtered using fastp v0.19.3 software [39] to remove adapters and low-quality reads (Q ≤ 20, N content > 10%). Clean reads were assembled into transcripts using Trinity v2.11.0 software [40], and assembled transcripts were clustered using Corset v1.09 [41]. Differentially expressed genes (DEGs) were identified using DESeq2 1.22.2 [42] with thresholds of |log_2_(fold change)| > 1 and FDR < 0.05, consistent with the transcript-level volcano plots in Figure 7. DESeq2-adjusted probabilities were interpreted within the false-discovery-rate framework [43]. GO and KEGG enrichment analyses were performed using GOATOOLS https://github.com/tanghaibao/goatools (accessed on 15 November 2025) [44] and KOBAS https://kobas.cbi.pku.edu.cn/ (accessed on 15 November 2025) [45], respectively. Transcriptomic enrichment results are displayed as Q values in Figure 7. A term appearing among the displayed annotations was not automatically interpreted as significant. The complete DEG lists are designated Supplementary Tables S1–S3.

**Figure 8:**
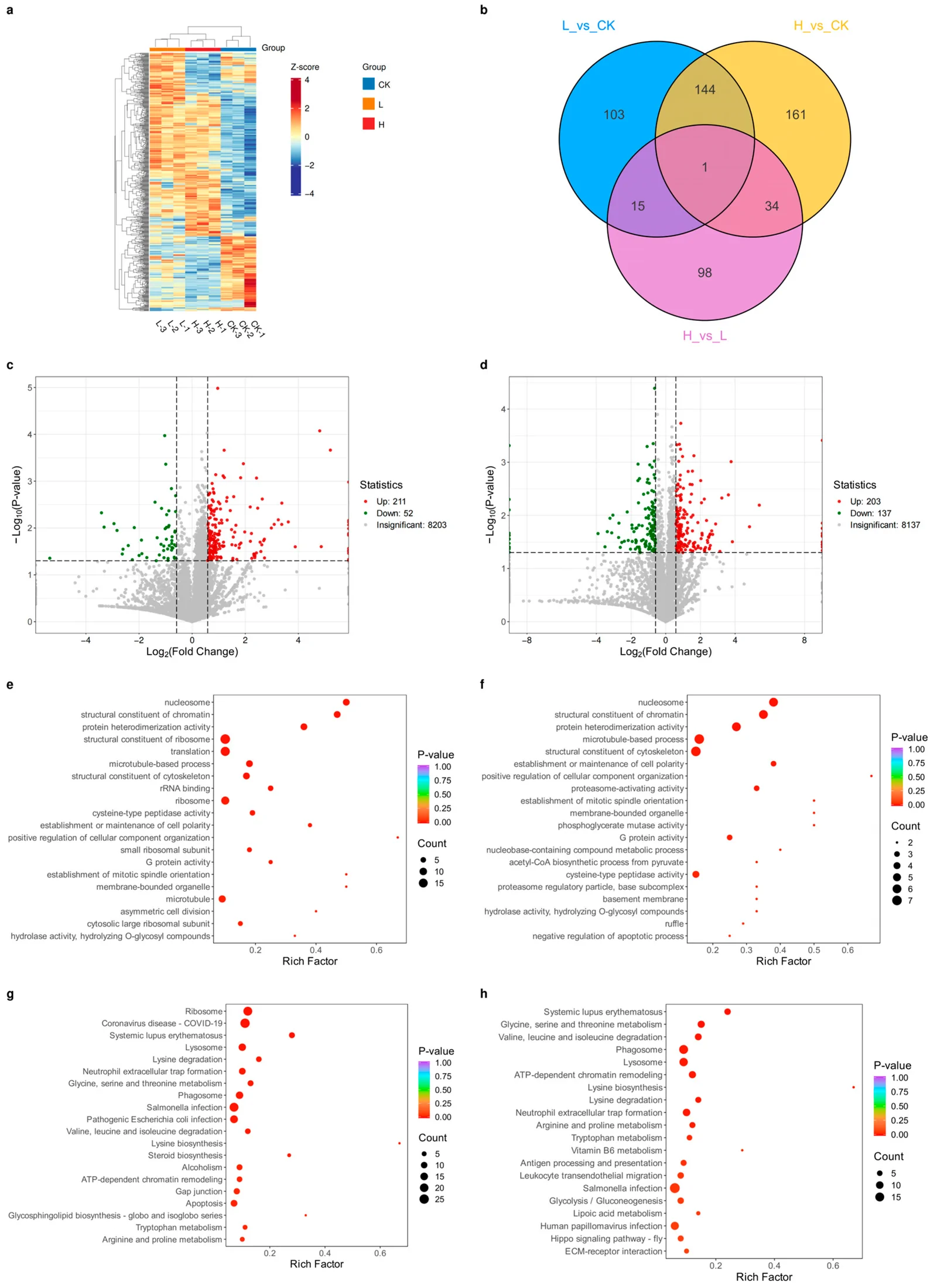
Proteomic profiling of planarians exposed to cigarette smoke extract. Note: (**a**) Hierarchical clustering heatmap of differentially expressed proteins (DEPs) across samples; (**b**) Venn diagram of DEPs among pairwise comparisons; (**c**) volcano plot of DEPs between the control (CK) group and the 1% CSE (L) group; (**d**) volcano plot of DEPs between the CK group and the 5% CSE (H) group; (**e**) GO enrichment analysis of DEPs (Control vs. 1% CSE); (**f**) GO enrichment analysis of DEPs (Control vs. 5% CSE); (**g**) KEGG pathway enrichment analysis of DEPs (Control vs. 1% CSE); (**h**) KEGG pathway enrichment analysis of DEPs (Control vs. 5% CSE). The volcano plots and enrichment panels report nominal *p*-values. These results are exploratory and are not presented as FDR-adjusted protein or pathway findings.

**Figure A2:**
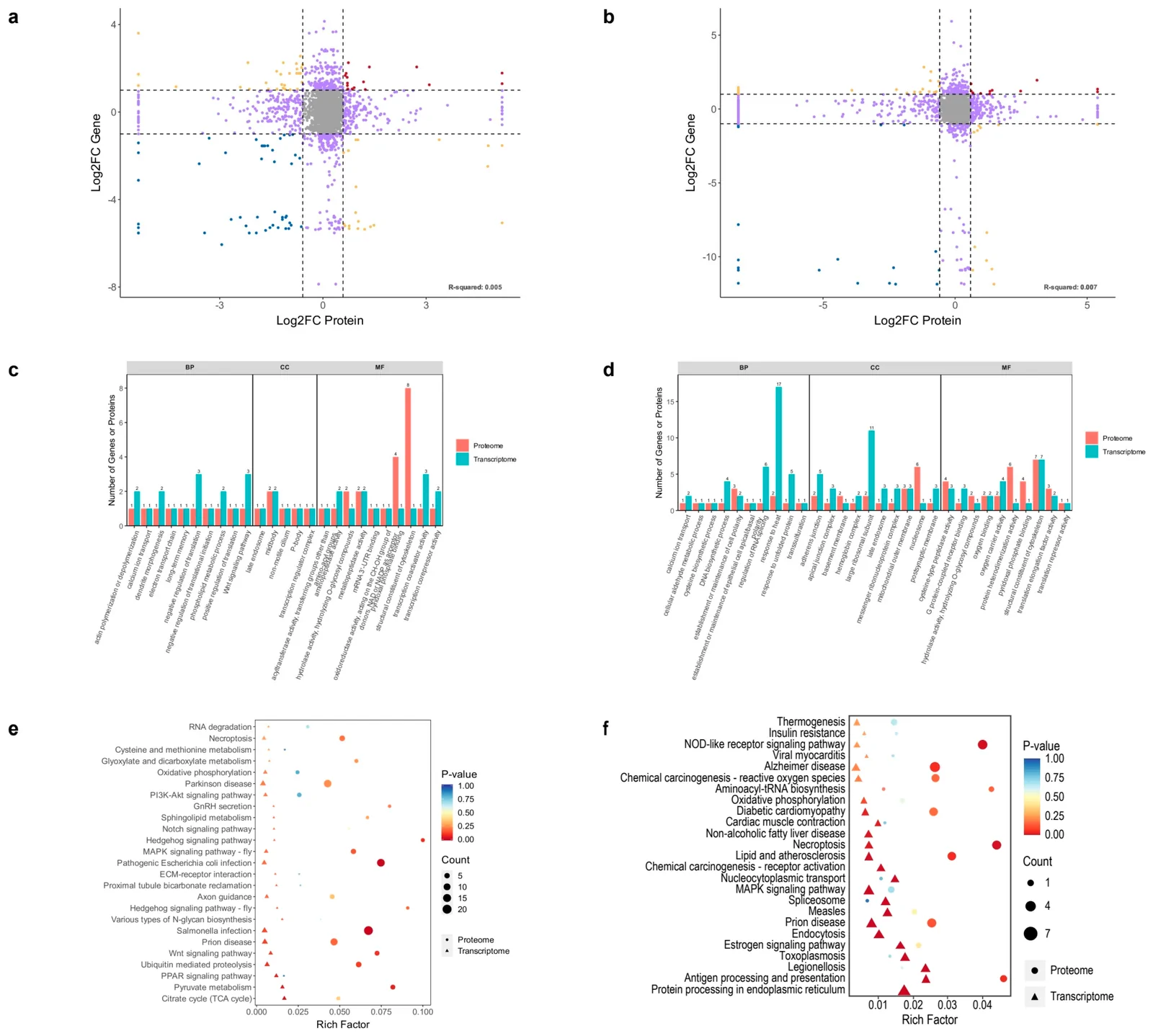
Joint analysis of transcriptomic and proteomic data. Note: (**a**) Nine-quadrant diagram of correlation between DEGs and DEPs (Control vs. 1% CSE); (**b**) nine-quadrant diagram of correlation between DEGs and DEPs (Control vs. 5% CSE); (**c**) comparison of GO annotations of DEGs and DEPs (Control vs. 1% CSE); (**d**) comparison of GO annotations of DEGs and DEPs (Control vs. 5% CSE); (**e**) comparison of KEGG pathway annotations of DEGs and DEPs (Control vs. 1% CSE); (**f**) comparison of KEGG pathway annotations of DEGs and DEPs (Control vs. 5% CSE). KEGG panels report nominal *p*-values. Shared annotation labels do not establish significant enrichment in both datasets and pathway activation.

Proteomic samples were collected after 72 h of exposure to 1% and 5% CSE, with Montjuïc water as the control and three biological replicates per group, at the same exposure endpoint as the transcriptomic samples. Total proteins were extracted from the paired protein aliquots described above, each originating from a pool of 10 animals, using RIPA lysis buffer, reduced with DTT, alkylated with IAA, and digested with trypsin (protein:trypsin = 50:1) at 37 °C overnight. Peptides were desalted using C18 solid-phase extraction columns and analyzed by LC-MS/MS (Vanquish Neo UHPLC coupled with Orbitrap Astral, Thermo Fisher Scientific, Waltham, MA, USA). Data were searched using DIA-NN v1.8.1 software [46] with library-free mode, and proteins were quantified using the MaxLFQ algorithm [47]. The differential protein sets shown in Figure 8 were screened using nominal *p* < 0.05 together with the fold-change filter displayed in the volcano plots. These protein comparisons were treated as exploratory rather than as FDR-controlled findings. Protein functional annotation was performed using InterProScan (v5.62-94.0) [48] and KAAS (KEGG Release 107.0) [49], and GO enrichment analysis was performed using topGO [50]. The proteomic GO and KEGG displays in Figure 8 report nominal *p*-values, as do the joint KEGG plots in Figure A2. The protein screening criterion remains nominal *p* < 0.05, as stated above.

### 2.11. Targeted Gene Expression and Immunoblotting Methods

**Figure 9:**
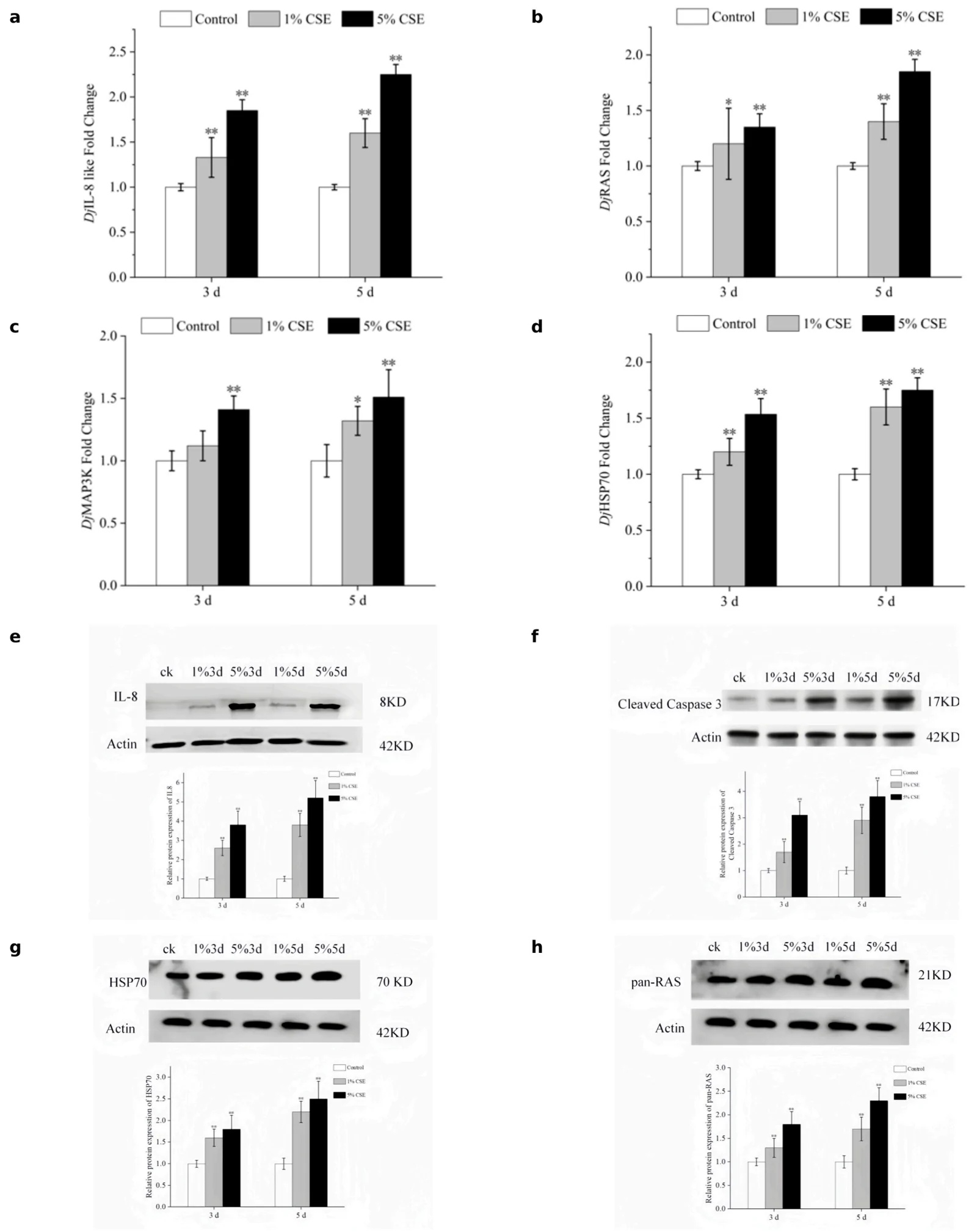
Targeted analysis of selected stress-response and MAPK-associated markers. Note: (**a**) Relative transcript expression of *DjIL-8-like*; (**b**) relative transcript expression of *DjRAS*; (**c**) relative transcript expression of *DjMAP3K*; (**d**) relative transcript expression of *DjHSP70*. Panels (**a**–**d**) show samples collected after 3 and 5 days of exposure to 1% and 5% CSE. (**e**–**h**) Relative intensities of immunoreactive bands assigned to IL-8, cleaved caspase-3, HSP70, and pan-RAS, respectively, normalized to β-actin. The pan-RAS signal does not distinguish individual RAS family members or measure RAS activation. Symbols indicate statistically significant differences at *p* < 0.05 (*) or *p* < 0.01 (**). Multiple-group comparisons used one-way ANOVA followed by Tukey’s HSD; comparisons involving two independent groups used independent-samples *t*-tests. Concentration groups were compared separately at each sampling time, using the independent batch summaries (*n* = 3). The original uncropped Western blot images corresponding to the immunoblot analyses are provided as Appendix A Figure A3 in the file “Raw Western blot images for indicated proteins”.

**Figure A3:**
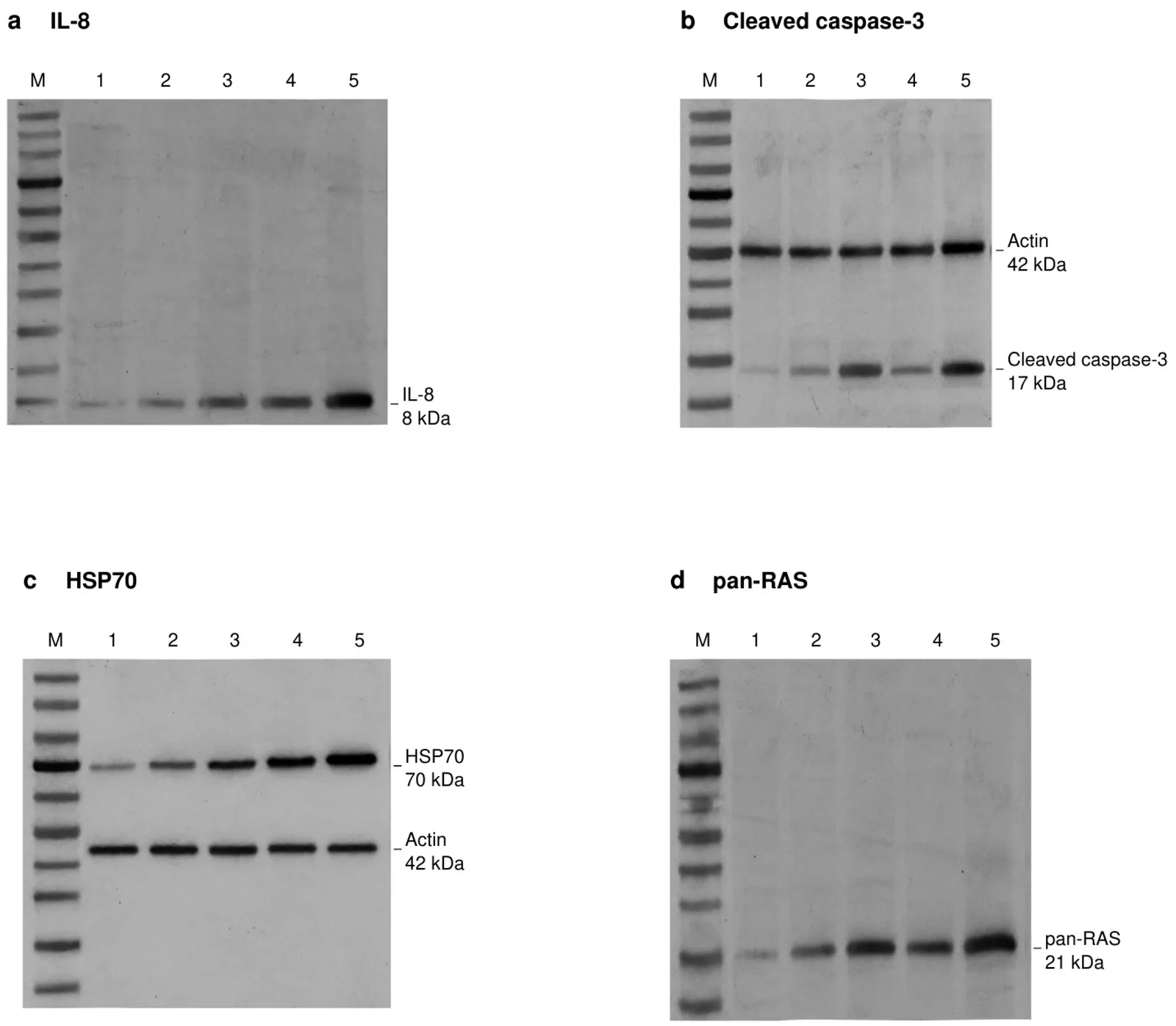
Full Western blot images for the indicated proteins. Note: (**a**) IL-8 (8 kDa), (**b**) cleaved caspase-3 (17 kDa), (**c**) HSP70 (70 kDa), and (**d**) pan-RAS (21 kDa), corresponding to Figure 9e–h, respectively. Actin (42 kDa) is indicated in panels (**b**,**c**). Protein names and molecular masses follow the annotations supplied with the source images. M denotes the molecular-mass marker; sample lanes are numbered 1–5 from left to right in their original order. The complete supplied image frames, including the marker lanes and surrounding background, are retained. No brightness, contrast, gamma, sharpening, or band-specific adjustments were applied.

qPCR was used to examine selected transcripts (*DjMAP3K*, *DjRAS*, *DjHSP70*, *DjIL-8-like*) after 3 and 5 days of exposure to 1% and 5% CSE, with Montjuïc water as the control, as described in Section 2.8 (Figure 9a–d). Western blotting was performed to examine selected protein signals (pan-RAS, HSP70, IL-8, and cleaved caspase-3) according to Ziman and Oviedo [51]. Each immunoblot biological sample comprised 10 pooled animals, with three independent biological replicates per condition. Briefly, protein samples were prepared with SDS-PAGE Sample Loading Buffer (5X; P0015, diluted to 1X; Beyotime) and separated by SDS-PAGE, transferred to PVDF membranes, blocked with 5% skim milk, incubated with primary antibodies (pan-RAS, rabbit, 81615-1-PBS; HSP70, mouse, 66183-1-Ig; IL-8/CXCL8, rabbit, 31533-1-AP; cleaved caspase-3, rabbit, 82707-13-RR; all 1:1000) at 4 °C overnight. Secondary detection used polymer HRP-conjugated goat anti-rabbit IgG (H+L) (RGAR011) for rabbit primary antibodies and goat anti-rabbit/mouse IgG (H+L) (RGAU011) for the mouse HSP70 primary antibody (both Proteintech; 1:5000, room temperature, 2 h). Bands were visualized using BeyoECL Star chemiluminescence reagent (P0018AS; Beyotime, Shanghai, China), and relative intensities were quantified using ImageJ with β-actin as the internal reference. Preliminary antibody assessments considered sequence conservation of the candidate targets and band positions. Mammalian cell lysate was included as a positive control in electrophoresis runs to assess antibody reactivity. These checks supported reagent selection but did not independently establish target specificity in *D. japonica*; the IL-8 and cleaved caspase-3 band assignments were interpreted cautiously. The manufacturer specifies that the pan-RAS antibody (81615-1-PBS) recognizes K-Ras, H-Ras, and N-Ras. This reagent does not distinguish individual RAS family members, and its mammalian reactivity does not independently validate the identity of the detected planarian protein. Total pan-RAS immunoreactivity is not a direct measurement of GTP-bound RAS activity. The original uncropped Western blot images are provided as Figure A3 in the file “Raw Western blot images for indicated proteins”.

### 2.12. Statistical Analysis

Data are presented as the mean ± standard deviation (SD). Statistical analyses were performed using SPSS 26.0 and GraphPad Prism 8.0. Independent exposure batches or independently prepared pooled samples were used as biological replicates; individual animals sharing a well and technical qPCR reactions did not increase the biological sample size. For the regeneration area ratio, biochemical measurements, qPCR, immunoblot endpoints, and batch-averaged H3P/TUNEL nuclear densities, multiple-group comparisons used one-way ANOVA, followed by Tukey’s HSD post hoc comparisons after a significant overall test. Comparisons involving two independent groups used two-sided independent-samples *t*-tests. Where multiple sampling times were examined, concentration groups were compared separately at each time, using independent batch summaries. The phenotype and mortality frequencies displayed in Figure 2b are descriptive; the statistical comparisons of regeneration concern the area ratio in Figure 2c. Individual nuclei and animals sharing a well were not treated as independent replicates for the density analyses. Time courses were described without inferring concentration-by-time interactions or differences between complete longitudinal trajectories. For qPCR, each gene at each sampling time was a separate comparison family. Tukey adjustment was applied within each family, not across all genes, endpoints, and sampling times. The *LC_50_* analysis and omics screening methods are described separately in Section 2.5 and Section 2.10. The symbols * and ** indicate *p* < 0.05 and *p* < 0.01, respectively.

## 3. Results

### 3.1. Component Analysis of CS and CSE by GC-MS

GC-MS analysis detected a total of 164 compounds across the CS and CSE samples under the analytical conditions used (Figure 1a,b). In the CSE profile, nicotine, phenols, nitriles, and amides accounted for 21.09%, 19.7%, 12.69%, and 8.17% of the detected peak area, respectively (Figure 1d,e). In the separately analyzed CS profile, nicotine, phenols, nitriles, esters, and aldehydes accounted for 39.88%, 3.58%, <1%, 14.03%, and 11.41%, respectively (Figure 1c,e). These values describe the relative peak-area profiles within each preparation. Because the sample preparation and injection methods differed, they do not support quantitative comparisons of compound concentrations between CS and CSE.

### 3.2. Acute Toxicity of CSE to D. japonica

The 96 h median lethal concentration (*LC_50_*) of CSE to *D. japonica* was 8.55% (95% confidence interval: 8.02–9.12%) based on probit analysis (Table 1). Mortality increased with concentration, reaching 100% at 11.0% CSE, whereas no mortality was observed at 6.0% CSE. This *LC_50_* value demonstrates the acute toxicity of CSE to freshwater planarians and informed the selection of lower concentrations for subsequent experiments.

### 3.3. Effects of CSE on Planarian Regeneration

CSE exposure at 1% and 5% impaired planarian regeneration at the assessed time points (Figure 2a). All control planarians completed regeneration within 7 days, whereas 1% CSE delayed regeneration without obvious phenotypic abnormalities. In the 5% CSE group, 2 out of 20 planarians died, and multiple phenotypic defects were observed, including absent eyespots, a single eyespot, head rounding, and body curling (Figure 2b). The blastema-to-total-body-area ratio was significantly lower in both CSE groups at 3, 5, and 7 days post-amputation, with a larger reduction in the 5% group (Figure 2c; *p* < 0.01). These results indicate impaired regeneration under the tested exposure conditions.

### 3.4. Effects of CSE on Oxidative Stress in Planarians

Oxidative stress-related responses varied across the tested CSE concentrations and time points. CSE at 1% significantly increased superoxide dismutase (SOD) and catalase (CAT) activities at 1–3 days, consistent with an antioxidant response. Higher concentrations (2% and 5%) inhibited SOD and CAT activities after 3 days (Figure 3a,b). Malondialdehyde (MDA) levels and the fluorescence signal used to assess ROS-related oxidation were significantly elevated in CSE-treated groups (*p* < 0.01), with 5% CSE producing the highest fluorescence signal (Figure 3c–e). These findings are consistent with altered redox homeostasis and increased lipid peroxidation.

### 3.5. Effects of CSE on Cholinergic Enzyme Activity and Neural Regeneration in Planarians

CSE exposure altered cholinergic enzyme activities in planarians. Choline acetyltransferase (ChAT) and acetylcholinesterase (AChE) activities were elevated in CSE-treated groups (1%, 2%, and 5%) at 1–5 days, with variation across concentrations and time points (Figure 4a,b; *p* < 0.01). Synapsin immunofluorescence showed no obvious structural changes in the nervous system of intact planarians (Figure 4c). In regenerating planarians exposed to 1% and 5% CSE, the images showed delayed closure of the brain ganglia and incomplete development of the ventral nerve cords at 5 days (Figure 4d). These qualitative observations suggest delayed neural remodeling during regeneration; enzyme activity measurements alone do not establish changes in acetylcholine turnover.

### 3.6. Effects of CSE on Cell Proliferation and Apoptosis

CSE exposure was accompanied by changes in proliferation- and apoptosis-related markers. Quantitative real-time PCR (qPCR) showed downregulation of the stem cell marker genes *DjpiwiA* and *DjpiwiB*, the proliferation marker *Djpcna*, and the cell-cycle regulator *DjCyclin B* at 1, 3, and 5 days of exposure (Figure 5a–d). The apoptosis-related genes *DjCaspase3* and *DjCaspase7* were upregulated at the same sampling times (Figure 6a,b). Immunofluorescence with an anti-phospho-histone H3 (H3P) antibody showed lower H3P-positive nuclear densities in both intact and regenerating planarians (Figure 5e–h). TUNEL staining showed higher densities of TUNEL-positive nuclei, consistent with increased DNA fragmentation (Figure 6c–f). Together, these observations are consistent with reduced mitotic activity and increased apoptosis-related responses at the sampled time points.

### 3.7. Transcriptome Analysis

Transcriptome sequencing of planarians exposed to 1% and 5% CSE for 72 h generated 581.15 million clean reads with Q30 ≥ 95.62% (Table 2). Under the screening criteria described in the Materials and Methods section, 74 differentially expressed genes (DEGs; 22 upregulated and 52 downregulated) were identified in the 1% CSE group, and 245 DEGs (68 upregulated and 177 downregulated) were identified in the 5% CSE group (Figure 7a–d). GO analysis highlighted metabolic terms such as protein initiator methionine removal in the 1% CSE group and stress-response terms such as protein refolding and response to heat in the 5% CSE group (Figure 7e,f). KEGG annotations included the citrate cycle and pyruvate metabolism at 1% CSE, and cardiac muscle contraction and prion disease at 5% CSE (Figure 7g,h). The latter are reference pathway labels and do not demonstrate cardiac tissue or prion disease in planarians.

### 3.8. Joint Analysis of Transcriptomic and Proteomic Data

Comparison of transcriptomic and proteomic changes showed limited overall concordance, with *R*^2^ = 0.005 for 1% CSE and *R*^2^ = 0.007 for 5% CSE (Figure A2a,b). Comparison of GO annotations identified terms represented in both datasets (Figure A2c,d). The displayed KEGG outputs included “MAPK signaling pathway-fly” at 1% CSE and “MAPK signaling pathway” at 5% CSE (Figure A2e,f). These reference annotations identify candidate responses for further study; their appearance in the plots does not establish concordant regulation, statistically significant enrichment in both datasets, or activation of MAPK signaling.

### 3.9. Targeted Analysis of Selected Genes and Proteins

qPCR analysis of selected stress-response and MAPK-associated transcripts (*DjIL-8-like*, *DjRAS*, *DjMAP3K*, and *DjHSP70*) showed upregulation at 3 and 5 days of CSE exposure (Figure 9a–d, respectively). *DjRAS* and *DjIL-8-like* were significantly elevated at all tested concentrations and time points, whereas *DjMAP3K* and *DjHSP70* were significantly upregulated at 5% CSE or after prolonged exposure (5 days). Western blotting showed increased signals for bands assigned to IL-8, cleaved caspase-3, HSP70, and pan-RAS (Figure 9e–h). Uncropped Western blot images are provided in Figure A3. These observations describe selected expression and immunoblot changes under CSE exposure; they do not directly measure MAPK pathway activation.

## 4. Discussion

The present study investigated the toxic effects and associated molecular responses of cigarette smoke extract (CSE) in the freshwater planarian *Dugesia japonica* at the whole-organism, biochemical, cellular, and molecular levels. Phenotypic observations, biochemical assays, immunofluorescence, and transcriptomic and proteomic profiling showed acute lethality, regeneration impairment, altered redox and cholinergic responses, and changes in proliferation- and apoptosis-related markers under CSE exposure. MAPK-related molecular changes were considered candidate stress responses rather than evidence of a demonstrated mechanism. These findings characterize the effects of the tested CSE preparation on planarians.

GC-MS analysis detected 164 compounds across CS and CSE under the analytical conditions used (Figure 1a–e). Nicotine accounted for 21.09% of the detected CSE peak area and is a well-documented component of tobacco smoke [2,52]. Phenols and nitriles were also present in the CSE profile. Because CS and CSE were prepared and introduced into the GC-MS system using different procedures, their relative peak areas cannot be used to infer quantitative enrichment or depletion. The CSE profile identifies potentially relevant constituents, but neither their absolute concentrations in the exposure medium nor their individual contributions to toxicity were determined. The biological effects therefore apply to the tested mixture rather than to any identified compound alone. Montjuïc water served as the exposure-medium control, whereas dichloromethane was restricted to separate GC-MS sample preparation. The water control does not isolate smoke-induced changes in pH, osmolarity, or other physicochemical properties. Thus, the observations concern the complete aqueous preparation rather than the effects of dissolved constituents independently of those properties. Related CSE exposure studies have also used aqueous or culture-medium preparations [3,4,12], but these precedents do not establish chemical equivalence between preparations or direct comparability with human smoke inhalation.

Planarian regeneration relies on neoblast proliferation and differentiation [6,7,53]. CSE had an acute lethal effect, with a reported 96 h *LC_50_* of 8.55% (Table 1), and the tested concentrations (1% and 5%) impaired regeneration (Figure 2a–c). CSE at 1% delayed regeneration without obvious phenotypic abnormalities, whereas 5% CSE caused mortality and defects including head rounding, eyespot malformation, and body curling. The reduced blastema-to-total-body-area ratio is consistent with impaired regrowth, but body contraction, shrinkage, or changes in shape can affect the denominator. Consequently, the ratio does not independently measure absolute blastema growth. Two treatment concentrations do not establish a full dose–response relationship, and systemic toxicity at 5% CSE may contribute to the regenerative defects. The absence of overt mortality at 1% CSE does not exclude sublethal systemic effects or establish a regeneration-specific mechanism. Separate reporting of blastema and total body areas, together with observations before overt tissue deterioration, would help distinguish these contributions. These results are consistent with other reports of impaired planarian regeneration following pollutant exposure [32,54]. Fewer H3P^+^ cells and altered expression of *DjpiwiA*, *DjpiwiB*, *Djpcna*, and *DjCyclin B* accompanied the regenerative changes (Figure 5a–h), together with increased TUNEL labeling and expression of apoptosis-related genes (Figure 6a–f). H3P labeling measures mitotic cells at the sampling time rather than total neoblast abundance. TUNEL positivity is consistent with DNA fragmentation [37], and cell death also occurs during normal regeneration [35,38]. Differences in regeneration stage and overall tissue condition therefore remain possible contributors to these cellular observations.

Oxidative stress has been reported in CSE-exposed cells [4,55]. Our results showed different antioxidant enzyme responses across the tested concentrations: 1% CSE increased superoxide dismutase (SOD) and catalase (CAT) activities at the reported early time points, whereas 2% and 5% CSE reduced their activities after 3 days (Figure 3a,b). Increased ROS-related fluorescence and malondialdehyde (MDA) levels were consistent with redox imbalance and lipid peroxidation (Figure 3c–e). DCFH-DA fluorescence does not identify a specific reactive species [56]. The enzyme increases may reflect an antioxidant response, but increased activity alone does not establish a protective benefit or hormesis. Oxidative stress-related changes have also been reported in planarians exposed to dibutyl phthalate [32]. ROS also have physiological roles: inhibiting ROS production impaired differentiation and brain regeneration in *Schmidtea mediterranea* [57]. ROS-related processes may contribute to the cellular responses observed here, but their causal relationship with mitotic changes, apoptosis, or regeneration impairment was not directly tested.

Planarians possess a simple yet functional nervous system and are useful for studying neural responses to toxicants [6,10,58]. CSE increased choline acetyltransferase (ChAT) and acetylcholinesterase (AChE) activities. ChAT catalyzes acetylcholine synthesis, whereas AChE catalyzes its hydrolysis [59,60,61]. Concurrent increases indicate changes in enzyme activity but do not establish the net change in acetylcholine concentration or turnover. In *D. japonica* exposed to organophosphorus pesticide mixtures, AChE inhibition was associated with some behavioral outcomes but not others [62]. Synapsin staining showed no obvious structural changes in intact planarians, while regenerating animals showed delayed brain ganglion closure and incomplete ventral nerve cord development (Figure 4c,d). These observations were qualitative, and quantitative neural morphometry was not performed. They therefore suggest delayed neural remodeling under CSE exposure but do not distinguish a specific neural effect from an overall delay in regeneration or systemic toxicity. Direct acetylcholine measurements and time-resolved neural morphometry would help clarify the functional consequences of these observations.

Transcriptomic and proteomic profiling provided information on molecular responses to CSE. Under the reported screening criteria, 74 and 245 differentially expressed genes (DEGs) and 263 and 340 differentially expressed proteins (DEPs) were identified in the 1% and 5% CSE groups, respectively (Figure 7 and Figure 8). The annotated terms included energy metabolism, translation, lysosome, and phagosome processes. Overall transcript–protein concordance was weak (*R*^2^ = 0.005 and 0.007 for 1% and 5% CSE, respectively; Figure A2a,b). In stimulated mouse dendritic cells, mRNA dynamics and protein synthesis and degradation contributed to changes in protein abundance [63]. Differences in RNA and protein turnover, translation, and protein processing can affect the timing and magnitude of abundance changes. Sequencing and mass spectrometry also differ in detection sensitivity, dynamic range, and quantification. The paired pooled-sample design is described in Section 2.10. Extraction and processing variation may contribute to weak correspondence. Both molecular datasets were collected at 72 h. A single sampling endpoint cannot resolve temporal differences, while whole-animal measurements combine different cell populations and may obscure cell-specific relationships. These are possible contributors, but the present analysis does not distinguish among them. Annotation overlap alone does not establish coordinated molecular regulation, and the joint interpretation remains exploratory. Transcriptomic plots report FDR/Q values, whereas proteomic and joint KEGG plots report nominal *p*-values; the latter do not demonstrate FDR-controlled pathway enrichment. Matched-pair counts and directional concordance would further clarify agreement. MAPK-related annotations and selected expression changes were therefore treated as candidate stress responses rather than a principal toxic mechanism. Targeted qPCR showed increases in *DjMAP3K*, *DjRAS*, *DjHSP70*, and *DjIL-8-like*, and immunoblot signals assigned to pan-RAS, HSP70, IL-8, and cleaved caspase-3 also increased (Figure 9a–h). These markers are not specific readouts of MAPK activation, and antibody specificity in *D. japonica* requires validation. In particular, the pan-RAS signal does not identify a specific RAS family member or establish its activation state. No validated quantitative measurement of ERK, JNK, or p38 phosphorylation or conclusive functional perturbation of MAPK signaling is included in this study. Preliminary attempts with phospho-specific antibodies and inhibitor exposure did not yield interpretable functional evidence under the conditions tested. ERK phosphorylation and pharmacological perturbation have been studied in planarians [64], so these practical difficulties do not imply that such measurements are generally infeasible. Inhibitor effects on regeneration alone cannot distinguish pathway-dependent effects from off-target toxicity; optimized intervention-only and combined-treatment controls and evidence of target engagement are needed. Thus, the data do not establish MAPK activation or MAPK-mediated apoptosis or regeneration impairment. Functional studies of ROS and ERK during regeneration in *Schmidtea mediterranea* [65] and MAPK-related responses in CSE-exposed mammalian cells [66] provide a basis for further testing, but do not establish the mechanism in this planarian CSE exposure model.

The biological sample size was three independent replicates for many endpoints. Pooling animals increased material available for measurement but did not increase the number of independent replicates. This sample size limits precision and sensitivity to smaller effects, particularly across multiple genes and sampling times; non-significant comparisons do not demonstrate an absence of biological effects. Preliminary concentration screening was qualitative and did not constitute a complete replicated dose–response analysis. The screening observations indicated weak and variable responses at 0.5% CSE, intermediate responses at 2%, and poor survival at 7% during the preliminary regeneration experiments, which made 7% unsuitable for the planned 7-day assay. These pilot observations were used for concentration selection and should not be treated as quantitative mortality estimates for the intact-animal acute toxicity experiment in Table 1.

The present results demonstrate toxicity to planarians under the tested CSE exposure conditions. Because environmental concentrations were not measured and the exposure was defined operationally, these results do not establish ecological risk or environmental exposure thresholds. Impaired tissue regeneration has also been reported in other cigarette-smoke exposure models [12], although differences in preparation, species, and exposure conditions limit direct comparison. Further studies using chemically characterized exposures would be needed to assess relevance to tobacco-related contaminants in aquatic environments. Calibrated measurements of representative constituents in the exposure medium and assessment of variation across independently prepared batches would improve exposure characterization. A new CSE stock was prepared every 24 h, but no dynamic OD_320_ measurements were made within that interval. Daily renewal reduces the time each preparation remains in use but does not demonstrate constituent stability within each renewal interval.

## 5. Conclusions

CSE exposure impaired regeneration and was accompanied by altered redox, cholinergic, and cellular responses in *Dugesia japonica*. MAPK-related changes represent a candidate stress response, but pathway activation and causal involvement were not established. These results characterize toxicity under the tested laboratory exposure conditions. Further work should chemically characterize the exposure, verify the proposed molecular responses, and test their functional relationship with regeneration impairment.

## Figures and Tables

**Table 1:** Mortality of planarians after 96 h of exposure to different CSE concentrations. Each row represents one independent exposure batch, with 10 animals per concentration per batch (30 per concentration across the three batches).

Exposure Batch	6.0%	7.0%	8.0%	9.0%	10.0%	11.0%
1	0	2	5	8	9	10
2	0	2	5	7	8	10
3	0	1	4	7	9	10

**Table 2:** Summary of RNA integrity and transcriptome sequencing quality metrics. RIN, RNA integrity number. Sample-specific RIN values were retrieved from laboratory notebook records. Q30 reflects base-call quality.

Sample	Clean Reads	Clean Bases (Gb)	Q30 (%)	RIN
CK-1	49,387,278	7.41	95.62	8.1
CK-2	61,988,382	9.3	95.79	8.3
CK-3	63,966,802	9.6	97.7	7.9
L-1	86,472,530	12.97	97.59	7.6
L-2	83,495,094	12.52	95.64	7.4
L-3	46,637,630	7.00	97.19	7.8
H-1	73,049,058	10.96	97.12	7.2
H-2	54,442,758	8.17	97.14	7.4
H-3	61,715,144	9.26	97.44	7.4

## Data Availability

The original uncropped Western blot images are provided as Appendix A Figure A3. The DEG workbooks are designated Supplementary Tables S1–S3. A complete DEP table is not included in the Supplementary Materials. Other raw data supporting the conclusions of this article will be made available by the authors on request.

## Supplementary Materials

The following supporting information can be downloaded at: https://www.mdpi.com/article/10.3390/biology15201821/s1, Table S1: Complete differentially expressed gene lists for all comparison sets; Table S2: Differentially expressed genes for the 1% CSE (L) vs. control (CK) comparison; Table S3: Differentially expressed genes for the 5% CSE (H) vs. control (CK) comparison.

## Abbreviations

The following abbreviations are used in this manuscript:

Abbreviation	Full Name
AChE	Acetylcholinesterase
ANOVA	Analysis of Variance
BCA	Bicinchoninic Acid
CAT	Catalase
ChAT	Choline Acetyltransferase
CS	Cigarette Smoke
CSE	Cigarette Smoke Extract
DEGs	Differentially Expressed Genes
DEPs	Differentially Expressed Proteins
DTT	Dithiothreitol
ECL	Enhanced Chemiluminescence
GC-MS	Gas Chromatography–Mass Spectrometry
GO	Gene Ontology
H3P	Phospho-Histone H3
IAA	Iodoacetamide
KEGG	Kyoto Encyclopedia of Genes and Genomes
*LC_50_*	Median Lethal Concentration
HSD	Honestly Significant Difference
MAPK	Mitogen-Activated Protein Kinase
MDA	Malondialdehyde
PBS	Phosphate-Buffered Saline
PBST	Phosphate-Buffered Saline with 0.1% Tween-20
qPCR	Quantitative Real-Time Polymerase Chain Reaction
RIN	RNA Integrity Number
ROS	Reactive Oxygen Species
SDS	Sodium Dodecyl Sulfate
SDS-PAGE	Sodium Dodecyl Sulfate Polyacrylamide Gel Electrophoresis
SOD	Superoxide Dismutase
TUNEL	Terminal Deoxynucleotidyl Transferase dUTP Nick End Labeling
VOCs	Volatile Organic Compounds

## Appendix A

**Table A1:** qPCR primer sequences.

Primer Name	Primer Sequence (5′–3′)
*β-DjActin-F*	ACCAGCAGATTCCATACCCA
*β-DjActin-R*	TTATGTTACGTTGCCCTCGA
*DjPiwiA-F*	TCAGCGGCTCATCATCCT
*DjPiwiA-R*	TCCGAAAATCCACATAAATAACATA
*DjPiwiB-F*	GAGTTAGGGCGTATGTTATTTTTTG
*DjPiwiB-R*	CAGGGACAGTCGTAGACAAGG
*DjCaspase3-F*	ACGGCAACAGTGAAAGATT
*DjCaspase3-R*	CTAAACAACAGGCAAAGAAA
*Djpcna-F*	GAATCGTCAAATGAAAGCG
*Djpcna-R*	GCAACAGTAATGGTGATAGCC
*DjCaspase7-F*	TTTACCCGAACGAGAAT
*DjCaspase7-R*	CGGATTAAATTTATCACCTCGTTC
*DjMAP3K-F*	CCGCTCAATCCGAAATCA
*DjMAP3K-R*	TAAACCGCTGCCTGAAAA
*DjRAS-F*	TTGTGGAGGCTCAAGTGC
*DjRAS-R*	ACGGAAGAACAAGGGAAC
*DjHSP70-F*	GTCGGGTATTCCTCCTGC
*DjHSP70-R*	TCTTTCCCTGTGCCTTTC
*DjIL-8-like-F*	TCTCTTCCTCCACCACCA
*DjIL-8-like-R*	CAGCACAGACCTCTCTCTTGAG
